# Identifying the oncogenic roles of FAP in human cancers based on systematic analysis

**DOI:** 10.18632/aging.204892

**Published:** 2023-07-24

**Authors:** Chao Ma, Shuaishuai Xi, He Sun, Meng Zhang, Yuanmin Pei

**Affiliations:** 1 School of Clinical Medicine, Weifang Medical University, Weifang 261053, Shandong, China; 2 Department of Vascular Surgery, Weifang Yidu Central Hospital, Weifang 262500, Shandong, China

**Keywords:** FAP, pan-cancer, prognosis, immune infiltration, immunotherapy

## Abstract

Background: Fibroblast activation protein-α (FAP) is a specific marker of cancer-associated fibroblasts (CAFs) and plays a crucial role in tumor development. However, the biological processes underlying FAP expression in tumor progression and tumor immunity have not been fully elucidated.

Methods: We utilized RNA-seq data from The Cancer Genome Atlas (TCGA) and Genotype-Tissue Expression (GTEx) to perform differential analysis of FAP expression in tumor tissues and matched-normal tissues. The relationship between FAP expression and clinical prognosis, DNA methylation, and tumor-infiltrating immune cells in pan-cancer was assessed using R Studio (version 4.2.1). Additionally, we employed gene set enrichment analysis (GSEA) and gene set variation analysis (GSVA) to investigate the biological functions and pathways associated with FAP expression.

Results: FAP exhibits high expression in most malignancies, albeit to a lesser extent in CESC, KICH, UCEC, SKCM, THCA, and UCS. Furthermore, FAP is either positively or negatively associated with the prognosis of several malignancies. In seven types of cancer, FAP expression is positively correlated with DNA methylation. CIBERSORT analysis revealed an inverse correlation between FAP expression and T cells, B cells, monocytes, and NK cells, while it exhibited a positive correlation with M0, M1, and M2 macrophages. Enrichment analysis further demonstrated that FAP modulates the cell cycle, epithelial-mesenchymal transition (EMT) process, angiogenesis, and immune-related functions and pathways.

Conclusion: Our findings indicate a close relationship between FAP expression and tumorigenesis as well as tumor immunity. FAP has the potential to serve as a diagnostic, prognostic, and immunotherapy marker.

## INTRODUCTION

Tumors are a leading global cause of death and pose a significant threat to public health [1]. In the field of medicine, various approaches to tumor management have emerged, aiming for individualization and precision [2, 3]. Immunotherapy has gained prominence as a major treatment for cancer, specifically through immune checkpoint blockade therapy [4]. The availability of public databases such as The Cancer Genome Atlas (TCGA) and GTEx has facilitated the identification of potential immunotherapy biomarkers by studying the correlation between gene expression, clinical survival, tumor-infiltrating immune cells (TIICs), and immunotherapy response [5].

FAP is selectively expressed on the surface of CAFs in various types of cancer [6–8]. It belongs to the family of dipeptidyl peptidases, exhibiting dipeptidyl peptidase and gelatinase activity. Structurally, FAP is composed of 760 amino acids and is a type II transmembrane serine protease. FAP elevation has been reported to contribute to cell proliferation, the EMT process, angiogenesis, and immunosuppression, thereby promoting tumor progression [6, 8].

Accumulating evidence suggests that individuals with upregulated FAP in tumors have worse clinical outcomes [9–13]. In mouse models of stomach adenocarcinoma (STAD), FAP-positive CAFs significantly contribute to cell proliferation and exhibit reduced sensitivity to anti-PD1 therapy [9]. In colon adenocarcinoma (COAD), elevated FAP accelerates malignant tumor progression by inducing resistance to immunotherapy through the reduction of immune cell infiltration levels and the promotion of an immunosuppressive microenvironment *in vivo* [10].

However, the majority of research on the role of FAP in tumors has focused on a single type of cancer. There has been no systematic analysis of FAP in pan-cancer. Hence, we explored the relationship between FAP expression and patient prognosis based on the TCGA, cancer cell line encyclopedia (CCLE), GTEx databases. Additionally, we investigated the correlation of FAP expression with DNA methylation, immune infiltration levels in 36 cancers. Moreover, we also studied FAP gene co-expression with immune-associated genes in various tumors. The biological activities of FAP in malignancies were examined using GSEA and GSVA. Our studies confirmed that FAP could be a prognostic biomarker and immunosuppressor for numerous malignancies by influencing the infiltration levels of tumor immune cells. This work also sheds light on the function of FAP in immunotherapy for tumors.

## METHODS

### Data processing and differential expression analysis

RNA-seq and clinical data were obtained from the TCGA and GTEx databases using the UCSC Xena website (https://xenabrowser.net/datapages/). Data from each tumor cell line was downloaded from the CCLE database (https://portals.broadinstitute.org/ccle/). All RNA-seq data were log2 transformed. The differential analysis between tumors and matched normal tissues in 36 tumors was conducted using R Studio (version 4.2.1). The results of the analysis were visualized using the R package “ggplot2”.

### Relationship between FAP expression and prognosis, pathological stage

Survival and clinicopathological data were retrieved from the TCGA database. The correlation between FAP expression and prognostic indicators, including overall survival (OS), disease-specific survival (DSS), progression-free interval (PFI), and disease-free interval (DFI), was analyzed using the R packages “survival” and “survminer”. Furthermore, the optimal cut-off value for FAP expression was determined using the R package “survival”. The significance of groups with high and low FAP expression was assessed using the survfit function. Additionally, an analysis was conducted to examine the correlation between FAP expression and clinicopathological stage using R Studio. The results of the analysis were visualized using the R package “ggplot2”.

### ROC curve for FAP expression in different cancers

The receiver operating characteristic curve (ROC) describes the relationship between sensitivity and specificity [14]. Statistical analysis was conducted on clinical data from the TCGA database using the R package “pROC”. The results were visualized using the R package “ggplot2”.

### Correlation between FAP expression and immunity

Based on the transcriptional profiles of tumor samples, the abundance of tumor cells, stromal cells, and immune cells was assessed using the R package “ESTIMATE”. The association between FAP expression and stromal, immune, and ESTIMATE scores was evaluated using the Spearman method. The results were visualized utilizing the R package “ggpubr”.

Currently, the CIBERSORT database serves as the most commonly employed tool for analyzing immune cell infiltration [15]. It enables the assessment of the proportion and abundance of 22 immune cell types in the tumor microenvironment (TME). In this study, we reevaluated the infiltration scores of 22 immune cell types in various tumors by utilizing the R package “IOBR”. The association between FAP expression and immune infiltration scores was analyzed using the Pearson method. The outcomes were depicted using the R packages “gcookbook” and “ggplot2”. Subsequently, to examine the role of FAP expression in tumor immunity, we explored the correlation between FAP expression and immune-associated genes, such as MHC genes, immune activators, immunosuppressors, chemokines, and chemokine receptors, employing the Spearman method.

### Correlation between FAP expression and DNA methylation

DNA methylation plays a role in tumor progression by modulating the expression levels of crucial genes and affecting various biological behaviors [16–18]. The DNA methylation data (Illumina human methylation 450) was derived from the TCGA database. To investigate the association between FAP expression and gene promoter methylation in each tumor, the Spearman correlation coefficient was employed. Furthermore, the relationship between FAP expression and the clinical prognosis of tumor patients was assessed using the Kaplan-Meier (KM) survival curve, which was plotted using the R packages “survival” and “survminer”.

### GESA and GSVA

We conducted GSEA and GSVA analyses to investigate the biological functions associated with FAP expression in various tumor types. The gene set of function and pathway was obtained from the official GSEA website (https://www.gsea-msigdb.org/gsea/downloads.jsp).

### Immunotherapy prediction analysis

Growing evidence suggests that immune checkpoint inhibitors (ICIs) substantially enhance the survival of patients with tumors and have emerged as a hot topic of current research [19–21]. To validate the impact of FAP expression on the response to immunotherapy, we chose the IMvigor210 cohort (bladder urothelial carcinoma, BLCA) and the GSE78220 cohort (SKCM). In this study, the KM curve was utilized to demonstrate the association between FAP expression and prognosis, while the difference in the response rate to immunotherapy between groups with high and low FAP expression was assessed through a Chi-square test.

### Drug sensitivity analysis

The CellMiner database integrates transcriptional profiles and pharmacological data from 60 tumor cell lines that were published by the National Cancer Institute (NCI) [22], and we performed the analysis of the connection between FAP expression and IC50 value using the CellMiner database.

### Cell culture

HK-2 (normal renal tubular epithelial cell line), 769-P, and ACHN (clear cell renal carcinoma cell lines) were derived from the Cell Bank of the Chinese Academy of Sciences and grown in F12, MEM, and 1640, respectively, supplemented with 10% FBS and 1% penicillin-streptomycin.

### Real-Time quantitative PCR

Total RNA was extracted from cell lines using Trizol Reagent, followed by reverse transcription into cDNA according to the manufacturer’s protocol. This procedure was conducted to investigate the levels of FAP mRNA expression in kidney renal clear cell carcinoma (KIRC). Quantitative real-time polymerase chain reaction (RT-qPCR) was conducted using the SYBR Green Master Kit on a LightCycler 480 II instrument. The sequences of primers were as follows: FAP: F: AGACTTGGTCCTTTTCAACGGT, R: ACG ATTTTTACCCAAGTCTTCATT. β-actin: F: CCCAT CTATGAGGGTTACGC, R: TTTAATGTCACGCAC GATTTC.

### Statistical analysis

R software (version 4.2.1) was used for statistical analysis in this study. The correlations between variables were examined employing either Pearson’s or Spearman’s methods. For determining significance, a threshold of *P* < 0.05 was adopted.

### Availability of data and materials

The datasets used and/or analyzed during the current study are available from the corresponding author on reasonable request.

## RESULTS

### Differential analysis of FAP expression between tumor and normal tissues

**Figure 1:**
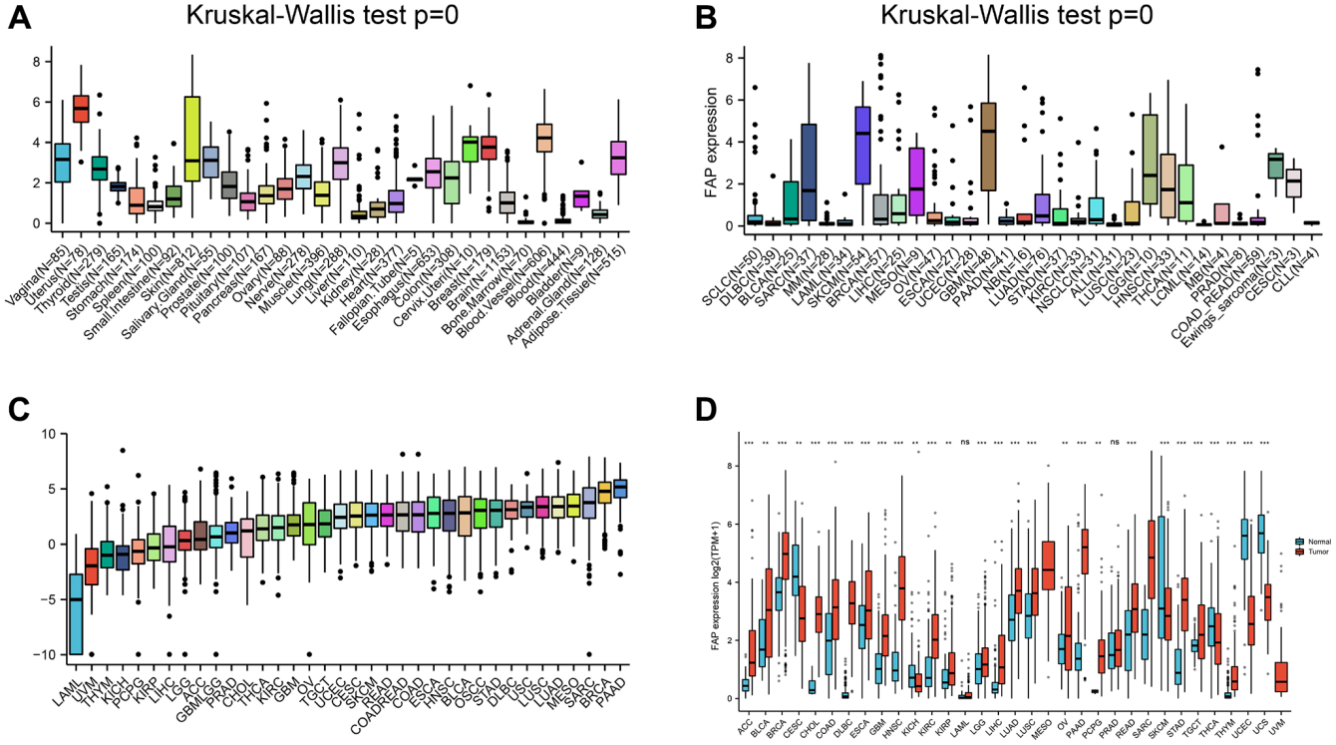
**Differential expression of FAP.** (**A**) FAP expression in normal tissues. (**B**) FAP expression in tumor cell lines. (**C**) FAP expression in 33 types of cancer. (**D**) Comparison of FAP expression between tumor and normal samples. ^*^P < 0.05, ^**^P < 0.01, ^***^P < 0.001.

Based on the GTEx database, we conducted a comprehensive analysis of FAP expression levels in 31 normal tissues. In general, FAP expression was relatively low in most normal tissues; however, it was significantly upregulated in the uterus, blood vessels, and cervix uteri tissues, which supports previous findings (Figure 1A). Furthermore, FAP protein, known as a specific biomarker for CAFs, was found to be expressed in various cancer cells and immune cells [23]. To illustrate this, Figure 1B presents the relative expression levels of FAP in 32 tumor cell lines obtained from the CCLE database. While the majority of tumor cell lines exhibited low FAP expression, human melanoma, brain glioma, and low-grade glioma cell lines showed elevated expression. Subsequently, we examined the expression levels of FAP in 36 tumor tissues and ranked them from low to high (Figure 1C). Notably, FAP expression levels were highest in pancreatic adenocarcinoma (PAAD) and lowest in acute myeloid leukemia (LAML). Additionally, by integrating TCGA and GTEx data, we thoroughly investigated the differential expression of FAP between 33 tumor and normal samples (Figure 1D). The results revealed that FAP was upregulated in 22 tumors and downregulated in six tumors. However, there were no significant differences in FAP expression in mesothelioma (MESO), sarcoma (SARC), and uveal melanoma (UVM), likely due to the limited availability of matched-normal tissues.

### Correlation between FAP expression and prognosis in different tumors

**Figure 2:**
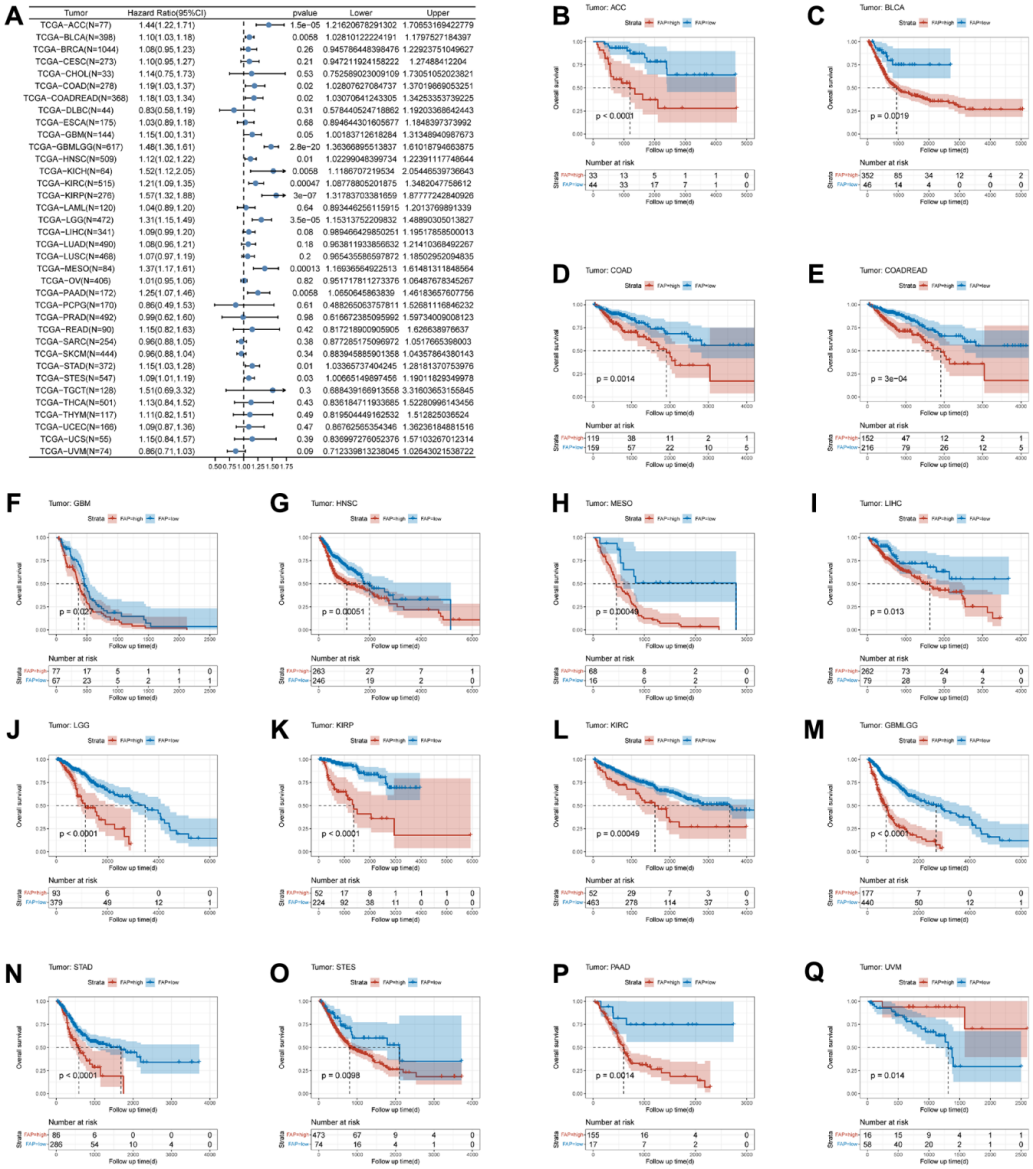
**Association between FAP expression and overall survival (OS).** (**A**) Forest plot of association of FAP expression and OS in pan-cancer. (**B**–**Q**) Kaplan-Meier analysis of the association between FAP expression and OS.

To investigate the correlation between the expression of FAP and prognostic indicators in tumor patients, namely OS, DFI, PFI, and DSS, we employed a Cox regression model and conducted KM survival analysis for each type of cancer. Statistical analysis was performed using the log-rank test. The results of the Cox regression model revealed a significant association between the level of FAP expression and OS in fourteen types of cancer, namely glioblastoma multiforme and lower-grade glioma (GBMLGG, HR = 1.48, *p*-value = 2.80E-20), kidney renal papillary cell carcinoma (KIRP, HR = 1.57, *p*-value = 3.00E-07), adrenocortical carcinoma (ACC, HR = 1.44, *p*-value = 1.50E-05), lower-grade glioma (LGG, HR = 1.31, *p*-value = 3.50E-05), mesothelioma (MESO, HR = 1.37, *p*-value = 1.30E-04), kidney renal clear cell carcinoma (KIRC, HR = 1.21, *p*-value = 4.70E-04), bladder urothelial carcinoma (BLCA, HR = 1.10, *p*-value = 5.80E-03), kidney chromophobe (KICH, HR = 1.52, *p*-value = 5.80E-03), pancreatic adenocarcinoma (PAAD, HR = 1.25, *p*-value = 5.80E-03), head and neck squamous cell carcinoma (HNSC, HR = 1.12, *p*-value = 0.01), stomach adenocarcinoma (STAD, HR = 1.15, *p*-value = 0.01), colon adenocarcinoma and rectum adenocarcinoma (COADREAD, HR = 1.18, *p*-value = 0.02), COAD (HR = 1.19, *p*-value = 0.02) and stomach and esophageal carcinoma (STES, HR = 1.09, *p*-value = 0.03) (Figure 2A). Therefore, FAP can be considered an independent risk factor for multiple types of cancer. The KM survival analysis further confirms that patients with high FAP expression have shorter OS in fifteen tumors (Figure 2B–2P). Conversely, UVM patients with high FAP expression exhibit longer OS, requiring further investigation (Figure 2Q).

**Figure 3:**
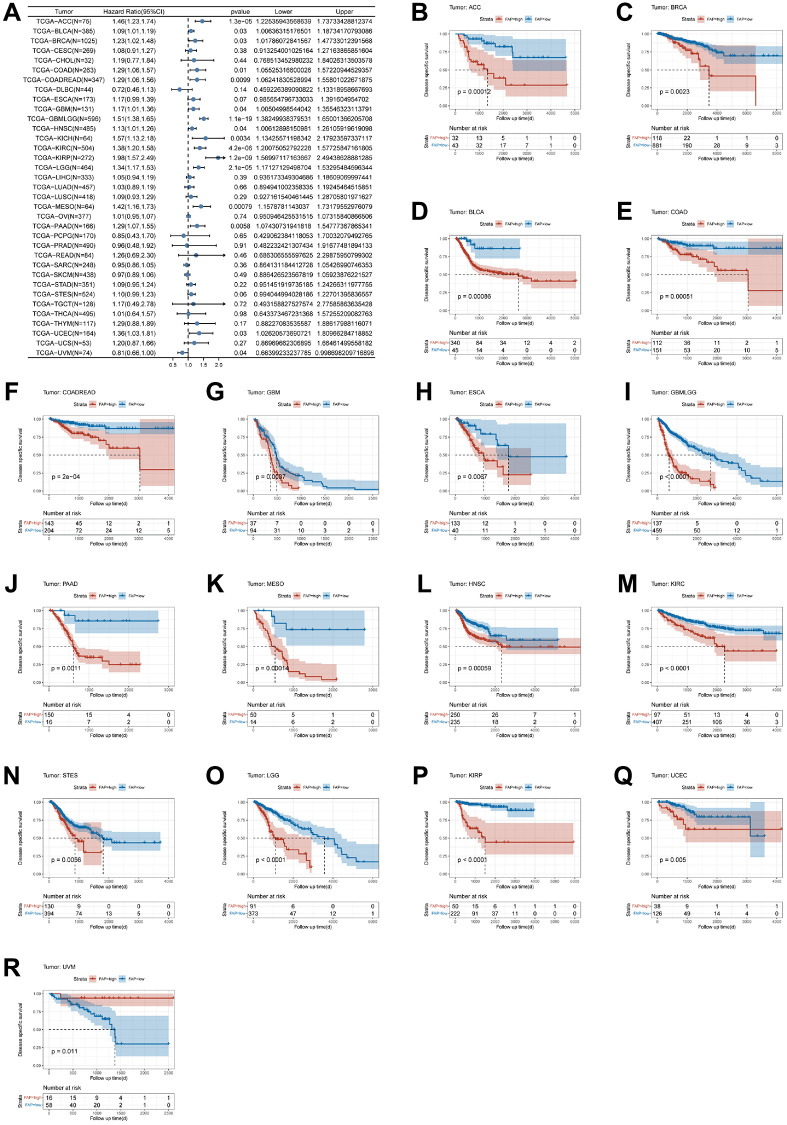
**Association between FAP expression levels and disease-specific survival (DSS).** (**A**) Forest plot of association of FAP expression and DSS in pan-cancer. (**B**–**R**) Kaplan-Meier analysis of the association between FAP expression and DSS.

Similarly, FAP expression in seventeen cancers was strongly linked to DSS (Figure 3A). The KM survival analysis revealed that patients with FAP overexpression have shorter DSS in sixteen tumors, including ACC (*p*-value = 0.00012), BRCA (*p*-value = 0.0023), BLCA (*p*-value = 0.00086), COAD (*p*-value = 0.00051), COADREAD (*p*-value = 2e-04), GBM (*p*-value = 0.0037), ESCA (*p*-value = 0.0067), GBMLGG (*p*-value < 0.0001), PAAD (*p*-value = 0.0011), MESO (*p*-value = 0.00014), HNSC (*p*-value = 0.00059), KIRC (*p*-value < 0.0001), STES (*p*-value = 0.0056), LGG (*p*-value < 0.0001), KIRP (*p*-value < 0.0001), and UCEC (*p*-value = 0.005), while UVM patients with high FAP expression have longer DSS times (*p*-value = 0.011) (Figure 3B–3R).

**Figure 4:**
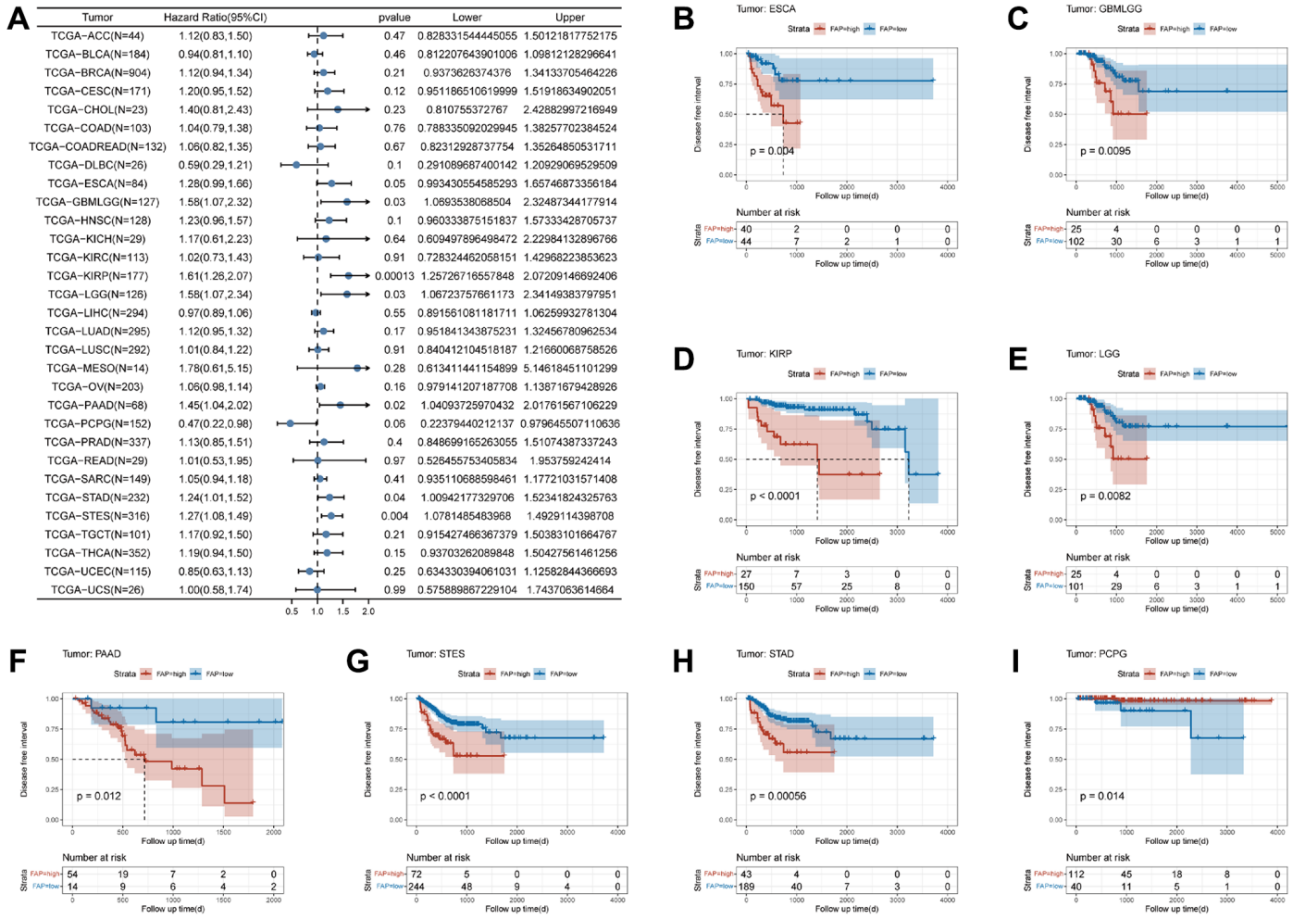
**Association between FAP expression levels and disease-specific survival (DFI).** (**A**) Forest plot of association of FAP expression and DFI in pan-cancer. (**B**–**I**) Kaplan-Meier analysis of the association between FAP expression and DFI.

Figure 4A showed that FAP expression was significantly related to DFI in six tumors, including KIRP (HR = 1.61, *p*-value = 0.00013), STES (HR = 1.27, *p*-value = 0.004), PAAD (HR = 1.45, *p*-value = 0.02), GBMLGG (HR = 1.58, *p*-value = 0.03), LGG (HR = 1.58, *p*-value = 0.03), and STAD (HR = 1.24, *p*-value = 0.04). Figure 4B–4H further demonstrated that individuals with high FAP expression have shorter DFI times, including ESCA (*p*-value = 0.004), GBMLGG (*p*-value = 0.0095), KIRP (*p*-value < 0.0001), LGG (*p*-value = 0.0082), PAAD (*p*-value = 0.012), STES (*p*-value < 0.0001), and STAD (*p*-value = 0.00056). In contrast, pheochromocytoma and paraganglioma (PCPG) patients with high FAP expression had longer DFI times (*p*-value = 0.014) (Figure 4I).

**Figure 5:**
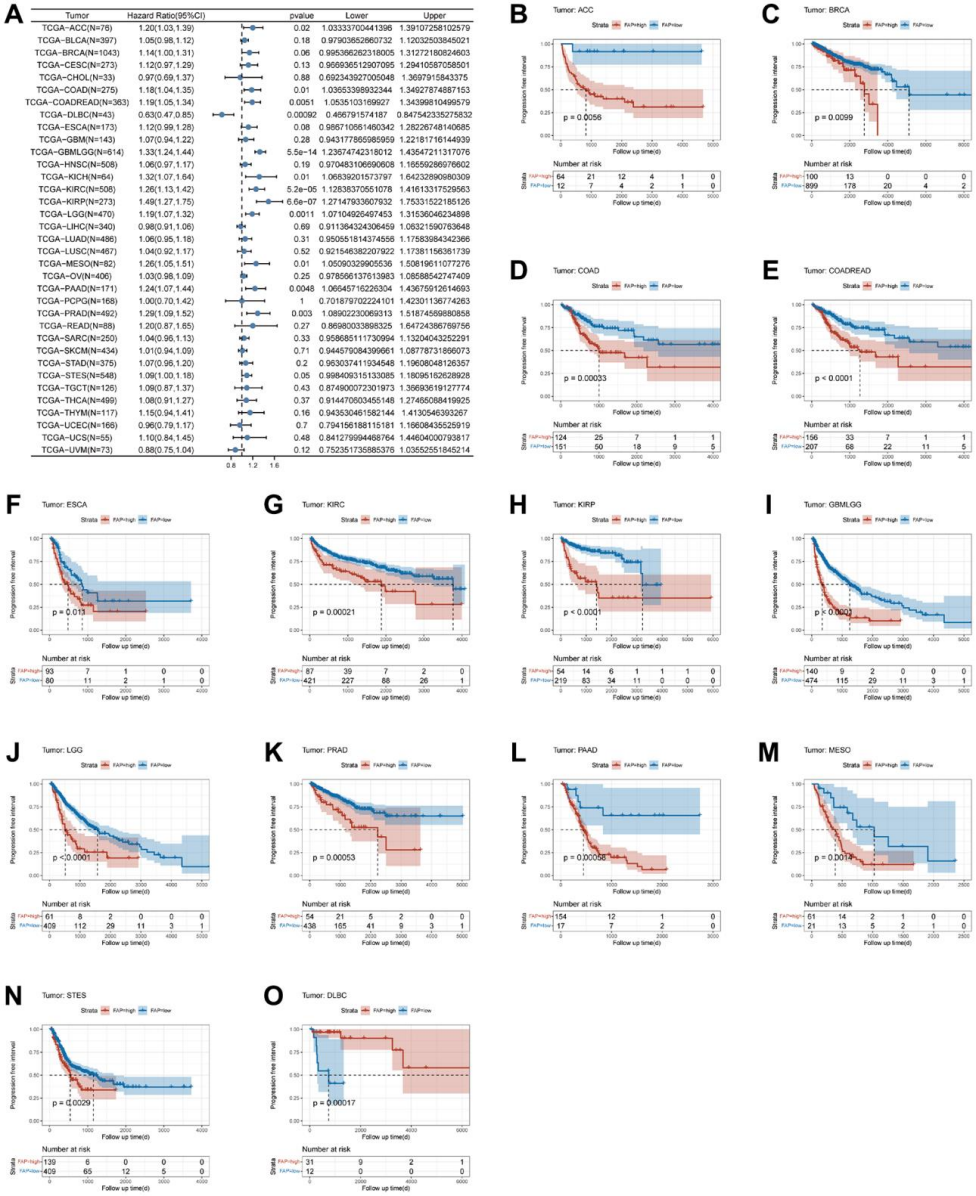
**Association between FAP expression and progression-free interval (PFI).** (**A**) Forest plot of association of FAP expression and PFI in pan-cancer. (**B**–**O**) Kaplan-Meier analysis of the association between FAP expression and PFI.

Furthermore, we conducted a comprehensive analysis of the correlation between FAP expression and PFI in various tumors. The forest plot revealed a positive association between FAP expression and poor prognosis in eleven tumors, such as GBMLGG, KIRP, KIRC, LGG, PRAD, PAAD, COADREAD, COAD, KICH, MESO, and ACC (Figure 5A). Additionally, the KM curve analysis further confirmed that high FAP expression was associated with poor PFI in thirteen tumors (Figure 5B–5N). However, FAP expression was found to be positively associated with improved prognosis in lymphoid neoplasm diffuse large B-cell lymphoma (DLBC, HR = 0.63, *p*-value = 0.00092) (Figure 5O).

### Correlation between FAP expression and pathological stage in various tumors

**Figure 6:**
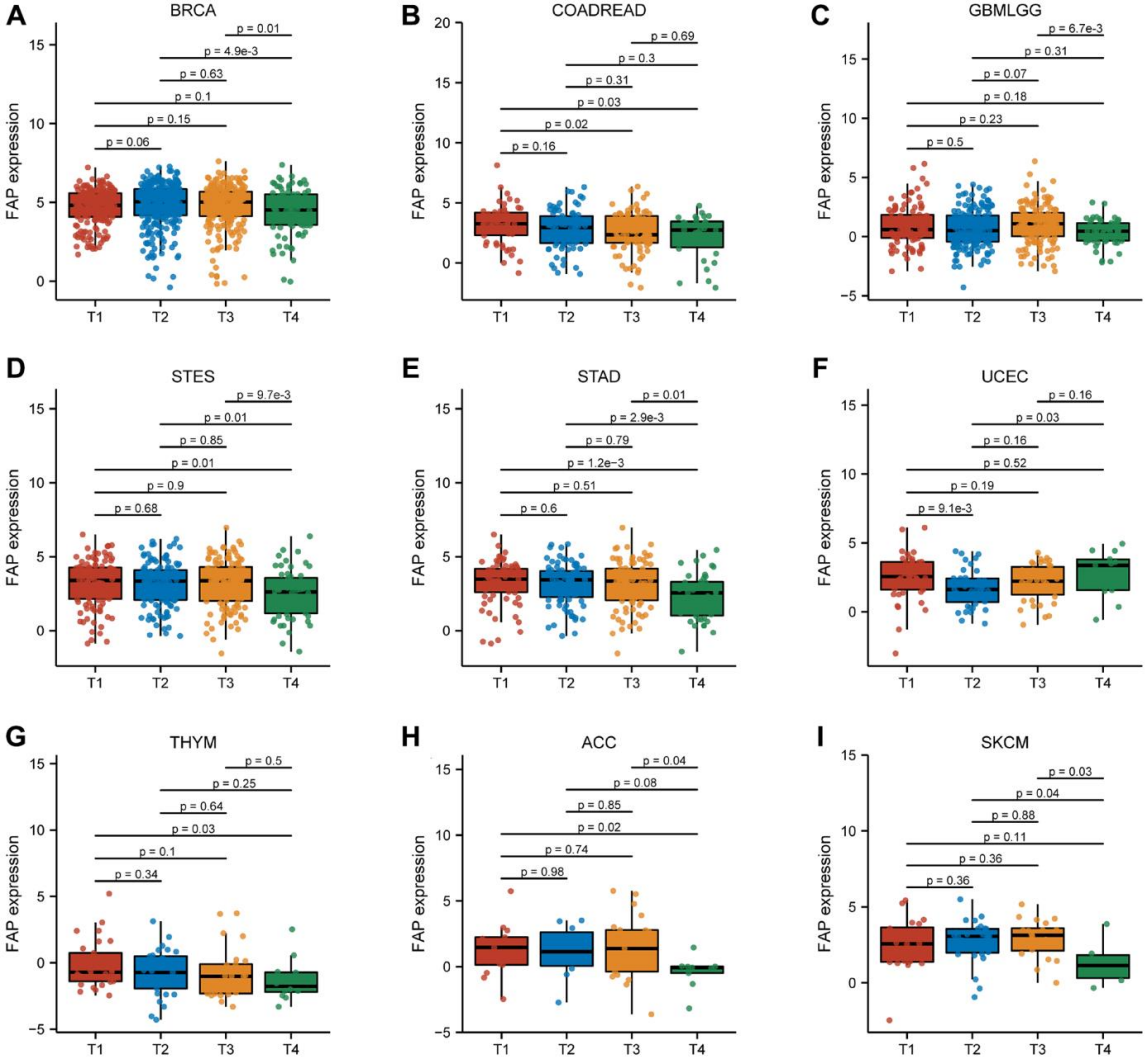
Association between FAP expression and T stage in (**A**) breast invasive carcinoma (BRCA), (**B**) colon adenocarcinoma/rectum adenocarcinoma esophageal carcinoma (COADREAD), (**C**) glioma (GBMLGG), (**D**) stomach and esophageal carcinoma (STES), (**E**) stomach adenocarcinoma (STAD), (**F**) uterine corpus endometrial carcinoma (UCEC), (**G**) thymoma (THYM), (**H**) adrenocortical carcinoma (ACC), (**I**) skin cutaneous melanoma (SKCM).

We conducted a study on the levels of FAP expression across various T stages. Figure 6 clearly demonstrates that FAP expression is substantially higher in T1, T2, and T3 stages compared to T4 stages in eight tumors. However, in UCEC, FAP expression in the T2 stage is significantly lower than in the T1 and T4 stages. It is important to note that FAP expression in other cancers shows no correlation with T stage.

**Figure 7:**
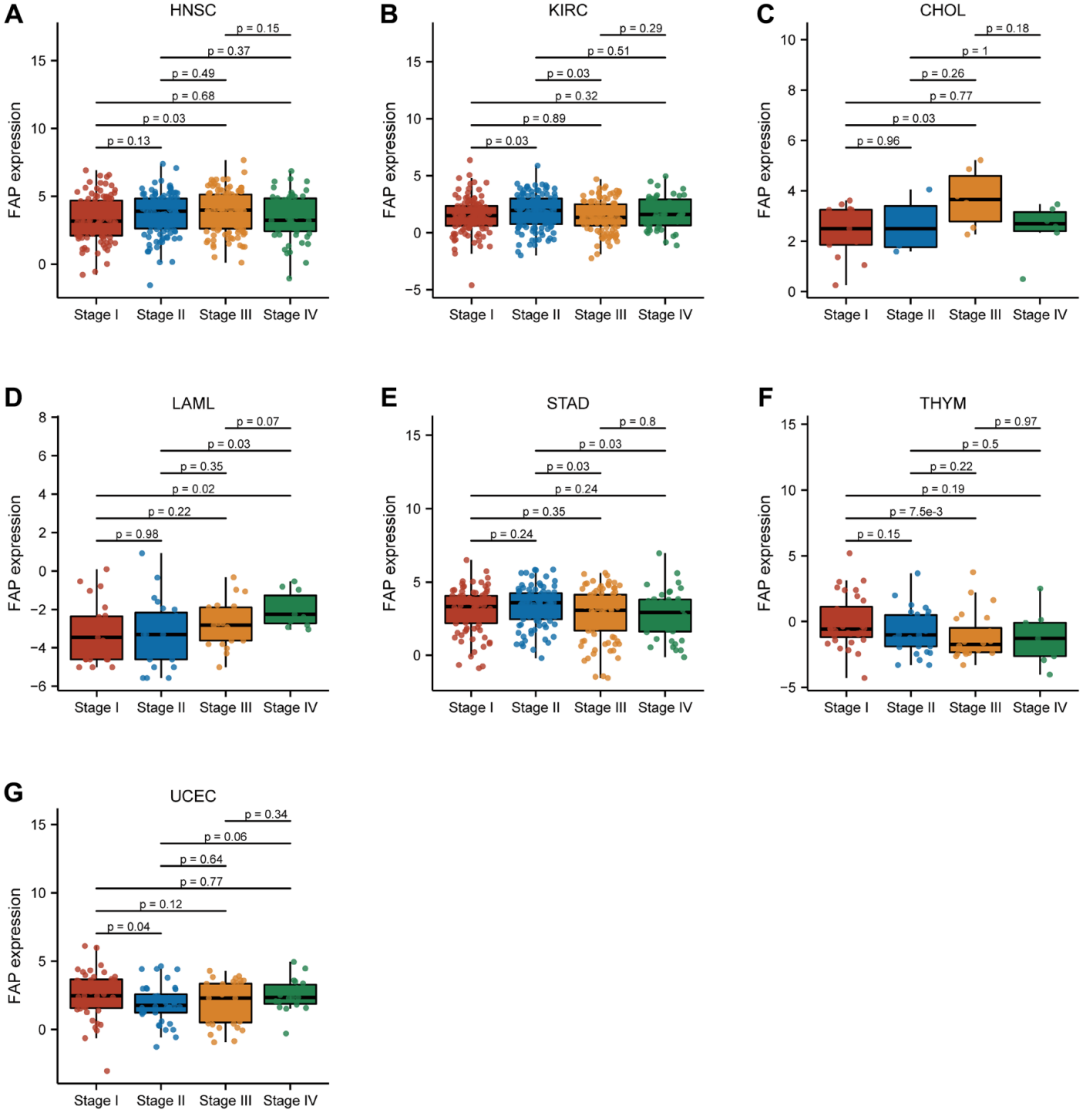
Association between FAP expression and pathological stage in (**A**) head and neck squamous cell carcinoma (HNSC), (**B**) kidney renal clear cell carcinoma (KIRC), (**C**) cholangiocarcinoma (CHOL), (**D**) acute myeloid leukemia (LAML), (**E**) stomach adenocarcinoma (STAD), (**F**) thymoma (THYM), (**G**) uterine corpus endometrial carcinoma (UCEC).

Furthermore, we also analyzed the association between FAP expression and pathological stage for each type of cancer. Our data reveals a significant correlation between FAP expression and pathological stage in seven tumors, including HNSC, KIRC, cholangiocarcinoma (CHOL), LAML, STAD, thymoma (THYM), and UCEC (Figure 7). In HNSC, CHOL, and LAML, FAP expression is significantly lower in stage I and II compared to stage III and IV. On the other hand, in STAD and THYM, FAP expression is significantly higher in stage I and II compared to stage III and IV. In KIRC, FAP expression in stage II is significantly higher than in stage I and III. In UCEC, FAP expression in stage I is substantially higher than in stage II.

### ROC curve for FAP expression in various cancers

The ROC curve reflects the diagnostic efficacy of FAP expression for each type of cancer. Fifteen tumors were screened, with an area under the curve (AUC) > 0.8 (Supplementary Figure 1). These included ACC (0.879), CHOL (0.978), DLBC (0.99), ESAD (0.899), GBM (0.839), GBMLGG (0.946), HNSC (0.900), KIRC (0.803), LIHC (0.810), OSCC (0.903), PAAD (0.947), STAD (0.905), THYM (0.913), UCEC (0.946), and uterine carcinosarcoma (UCS, 0.943).

### Relationship between FAP expression and the tumor microenvironment

**Figure 8:**
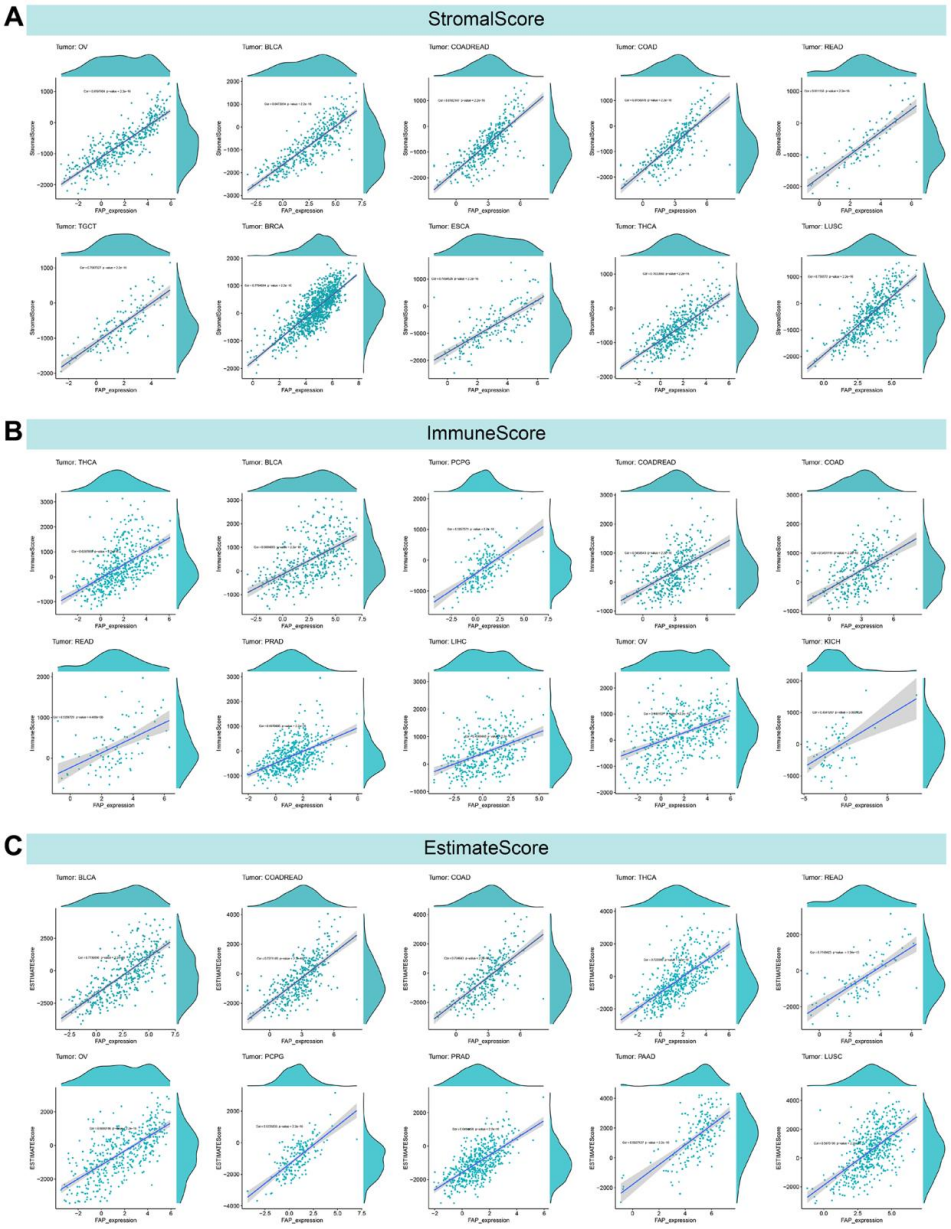
**Ten tumors with the highest correlation coefficients between FAP expression and the tumor microenvironment.** (**A**) Correlation between FAP and stromal scores in ovarian serous cystadenocarcinoma (OV), bladder urothelial carcinoma (BLCA), colon adenocarcinoma/rectum adenocarcinoma esophageal carcinoma (COADREAD), colon adenocarcinoma (COAD), rectum adenocarcinoma (READ), testicular germ cell tumors (TGCT), breast invasive carcinoma (BRCA), esophageal carcinoma (ESCA), thyroid carcinoma (THCA), lung squamous cell carcinoma (LUSC). (**B**) Correlation between FAP and immune scores in THCA, BLCA, pheochromocytoma and paraganglioma (PCPG), COADREAD, COAD, READ, prostate adenocarcinoma (PRAD), liver hepatocellular carcinoma (LIHC), OV, kidney chromophobe (KICH). (**C**) Correlation between FAP and ESTIMATE scores in BLCA, COADREAD, COAD, THCA, READ, OV, PCPG, PRAD, pancreatic adenocarcinoma (PAAD), LUSC.

The TME comprises tumor, stromal, and immune cells and is closely linked to cell proliferation, treatment resistance, metastasis, and angiogenesis [24–26]. The ESTIMATE algorithm was used to evaluate the correlation between FAP expression and StromalScore, ImmuneScore, and ESTIMATEScore. Figure 8 presents the top ten tumors exhibiting the strongest correlation between FAP expression and the TME. Supplementary Figures 2–4 depict the relationship between FAP expression and the TME in pan-cancer.

### Connection of FAP expression with TIICs

Accumulating evidence has demonstrated a strong correlation between TIICs and prognosis, immune response [27–29]. Our data revealed a close association between immune cell infiltration and FAP expression in the majority of malignancies. Eight tumors, namely BRCA (*N* = 16), BLCA (*N* = 13), PRAD (*N* = 13), THYM (*N* = 14), THCA (*N* = 16), OV (*N* = 15), LUSC (*N* = 12), and COADREAD (*N* = 12), exhibited significant associations with multiple immune cell types, thus warranting further investigation (Supplementary Table 1).

In the eight tumors, FAP expression exhibited an inverse relationship with the levels of infiltrating naive B cells, CD8 T cells, naive CD4 T cells, follicular helper T cells, resting NK cells, monocytes, and eosinophils. Conversely, FAP expression showed a positive correlation with the infiltration levels of M0 and M1 macrophages, as well as neutrophils. Notably, except for THCA, FAP expression exhibited a positive correlation with M2 macrophages in seven tumors.

**Figure 9:**
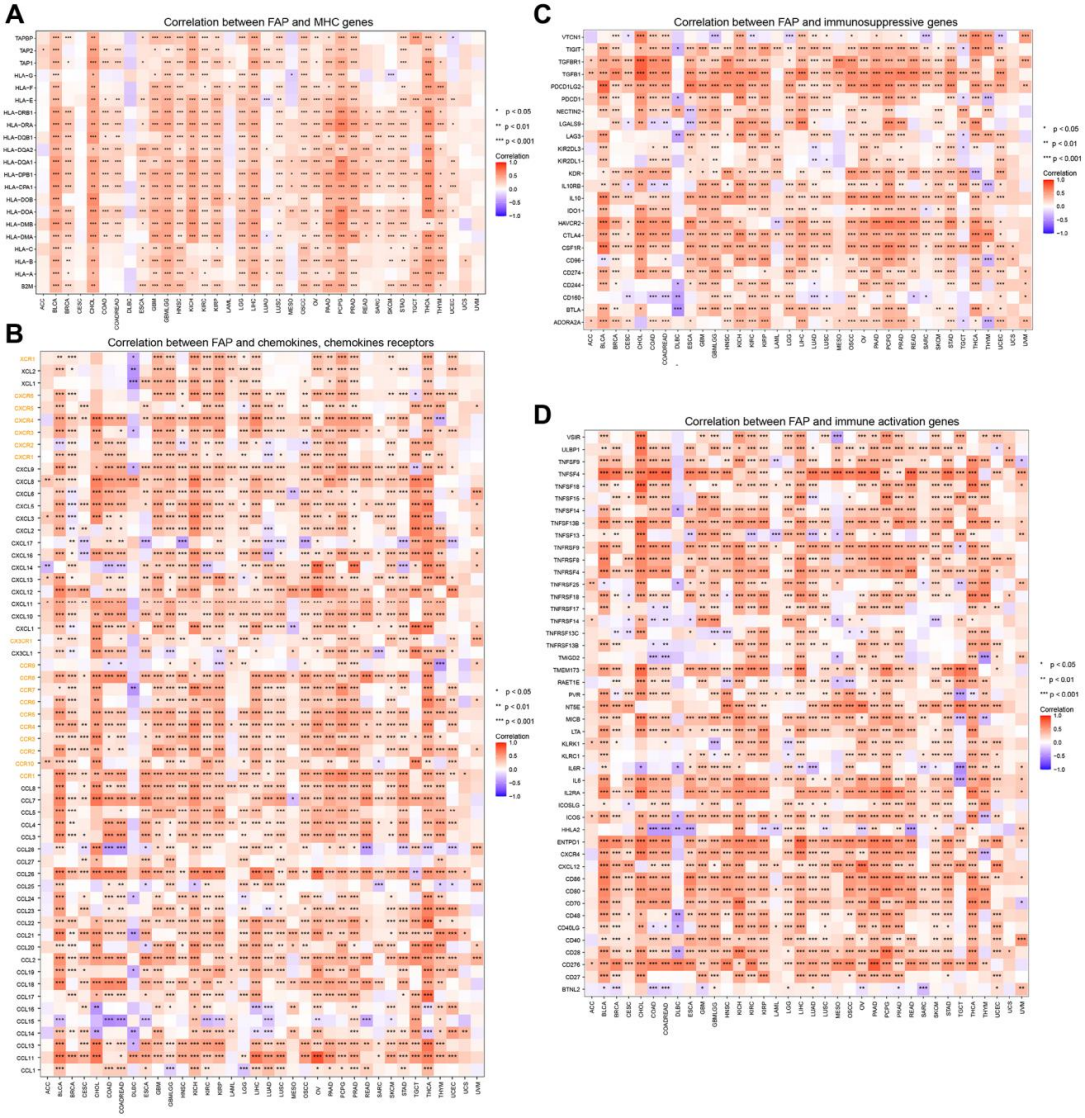
**Co-expression of FAP and immune-related genes.** (**A**) Correlation between FAP and MHC genes. (**B**) Correlation between FAP and chemokines, chemokines receptors, the yellow font represents chemokine receptors. (**C**) Correlation between FAP and immunosuppressive genes. (**D**) Correlation between FAP and immune activation genes. ^*^P < 0.05, ^**^P < 0.01, ^***^P < 0.001.

To further study the role of FAP expression in tumor immunity, an analysis of the connection between FAP expression and MHC genes, immune activators, immune suppressors, chemokines, and chemokine receptors was conducted in 36 tumors. The heatmap illustrates that most immune-related genes have a significant positive correlation with FAP expression across cancer types, except for DLBC (Figure 9A–9D). Supplementary Figure 5 depicts cancers with the strongest connection between FAP expression and infiltration levels of 22 immune cells; data for other malignancies can be found in Supplementary Table 2.

### Correlation of FAP expression with DNA methylation

**Figure 10:**
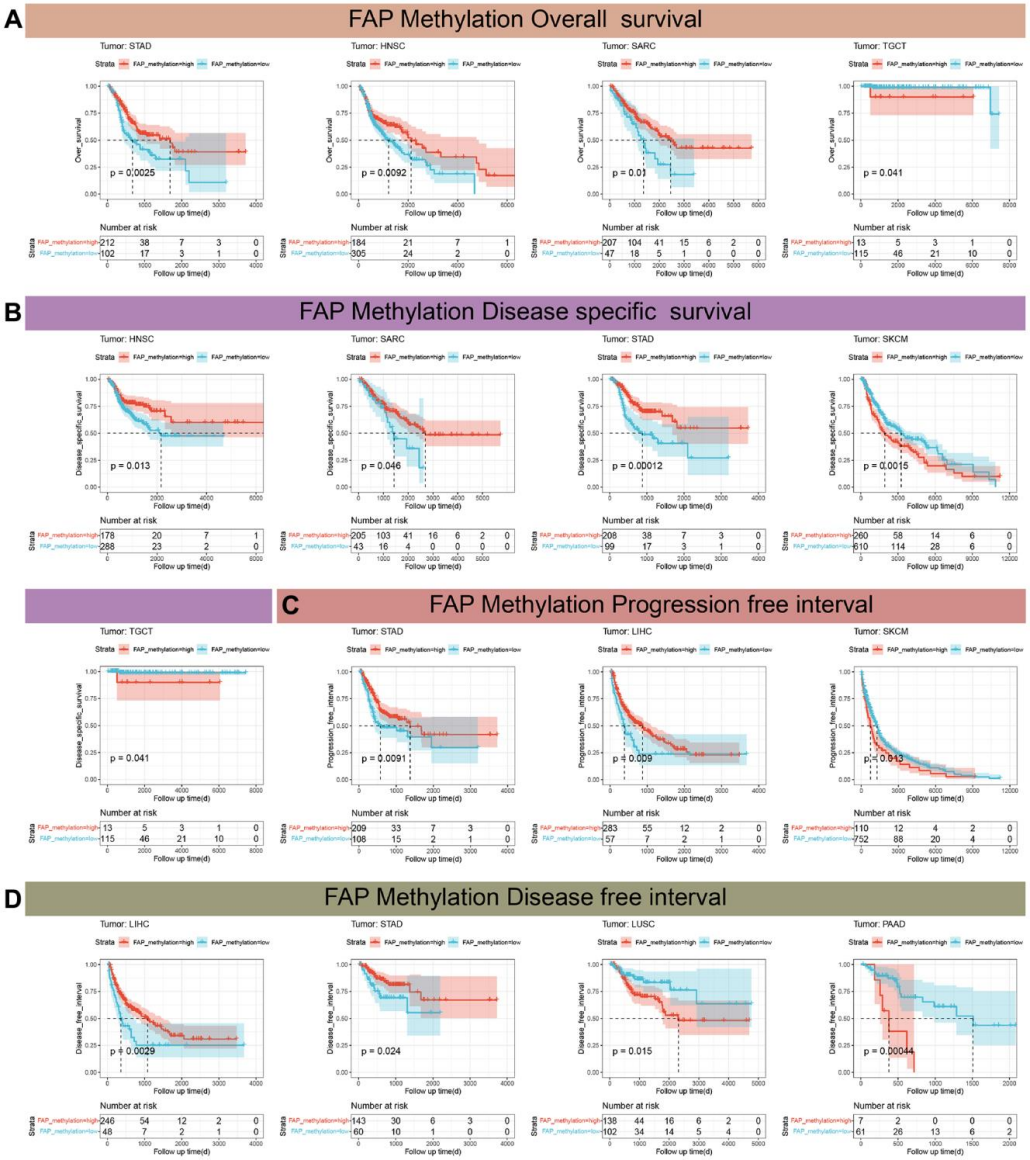
**Kaplan-Meier analysis of the association between gene promoter methylation and prognosis.** (**A**) Correlation between FAP methylation and OS in testicular germ cell tumors (TGCT), stomach adenocarcinoma (STAD), sarcoma (SARC), head and neck squamous cell carcinoma (HNSC). (**B**) Correlation between FAP methylation and DSS in STAD, SARC, TGCT, HNSC, skin cutaneous melanoma (SKCM). (**C**) Correlation between FAP methylation and PFI in STAD, SKCM, liver hepatocellular carcinoma (LIHC). (**D**) Correlation between FAP methylation and DFI in LIHC, STAD, lung squamous cell carcinoma (LUSC), pancreatic adenocarcinoma (PAAD).

Supplementary Figure 6 illustrates a significant association between FAP expression and DNA methylation in ten different tumors. Furthermore, we conducted additional analysis to investigate the impact of DNA methylation levels on the prognosis of patients with tumors. Figure 10A, 10B indicates that elevated FAP methylation levels were associated with longer OS and DSS in STAD, HNSC, and SARC. In TCGT, high FAP methylation levels were linked to shorter OS and DSS. Moreover, high FAP methylation levels were correlated with a shorter DSS and PFI in SKCM (Figure 10C). In STAD and LIHC, increased FAP methylation levels were associated with a longer PFI and DFI, while in LUSC and PAAD, high FAP methylation levels were related to a shorter DFI (Figure 10D).

### GSEA and GSVA

**Figure 11:**
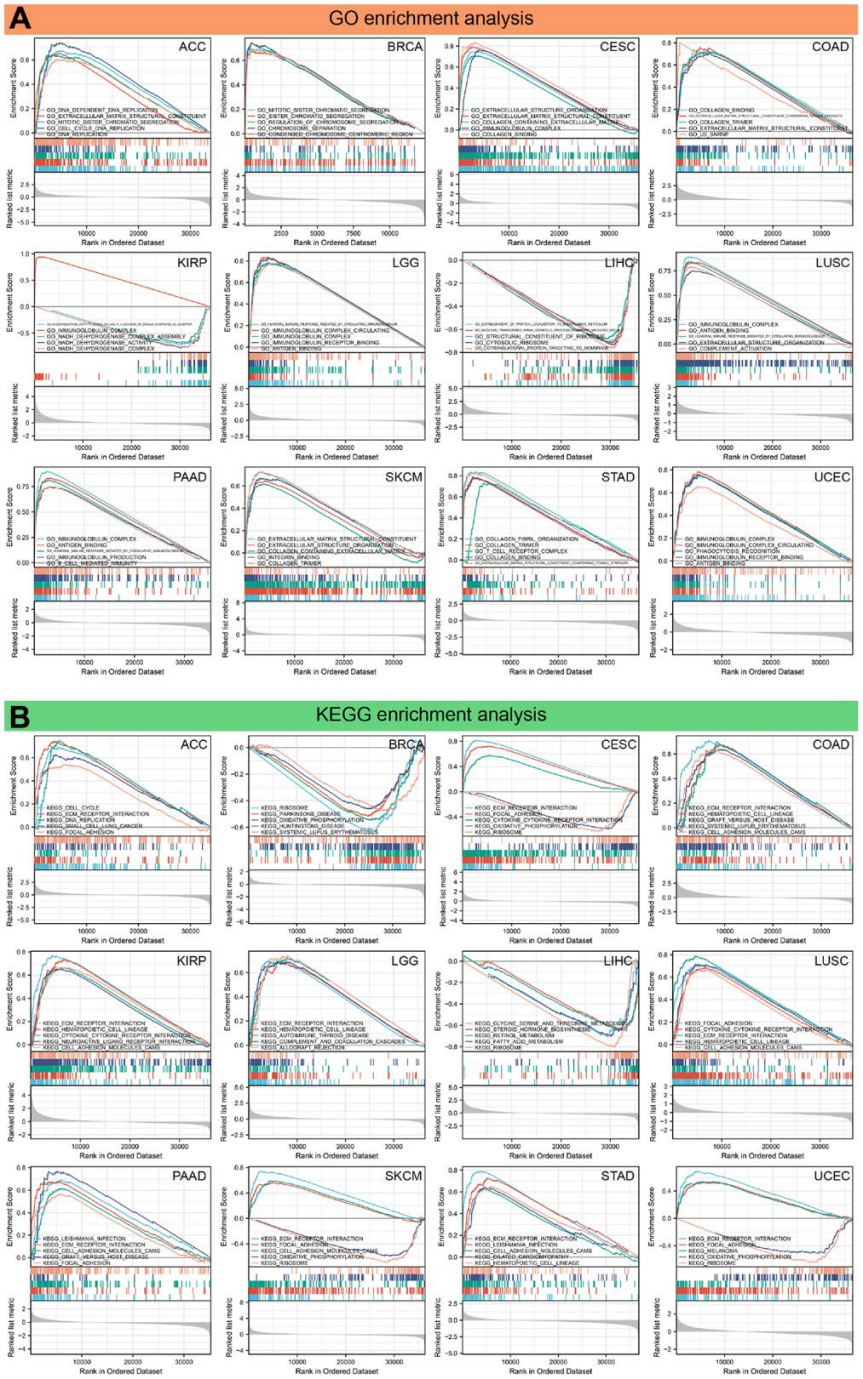
**Results of GSEA.** (**A**) GO functional annotation of FAP in various cancers. (**B**) KEGG pathway analysis of FAP in multiple cancers. Curves of different colors show different functions or pathways regulated in different cancers. Peaks on the upward curve indicate positive regulation and peaks on the downward curve indicate negative regulation.

To investigate the biological significance of FAP expression, GSEA and GSVA analyses were conducted to explore the biological processes involved in FAP expression. Figure 11A illustrates that FAP positively regulates cell proliferation, migration, immune, and energy metabolism-related functions in eleven tumors, except for LIHC.

Furthermore, KEGG analysis revealed that FAP positively regulates cell cycle, DNA replication, ECM receptor interaction, focal adhesion, cytokine and cytokine receptor interaction, cell adhesion molecules (CAMs), hematopoietic cell lineage, as well as immune-related pathways in ten tumors, except for BRCA and LIHC (Figure 11B). On the other hand, FAP is predicted to negatively regulate ribosome and oxidative phosphorylation in BRCA, CESC, LIHC, SKCM, and UCEC. Notably, FAP is predicted to hinder processes associated with energy metabolism, including fatty acid metabolism, glycine serine metabolism, threonine metabolism, and retinol metabolism.

**Figure 12:**
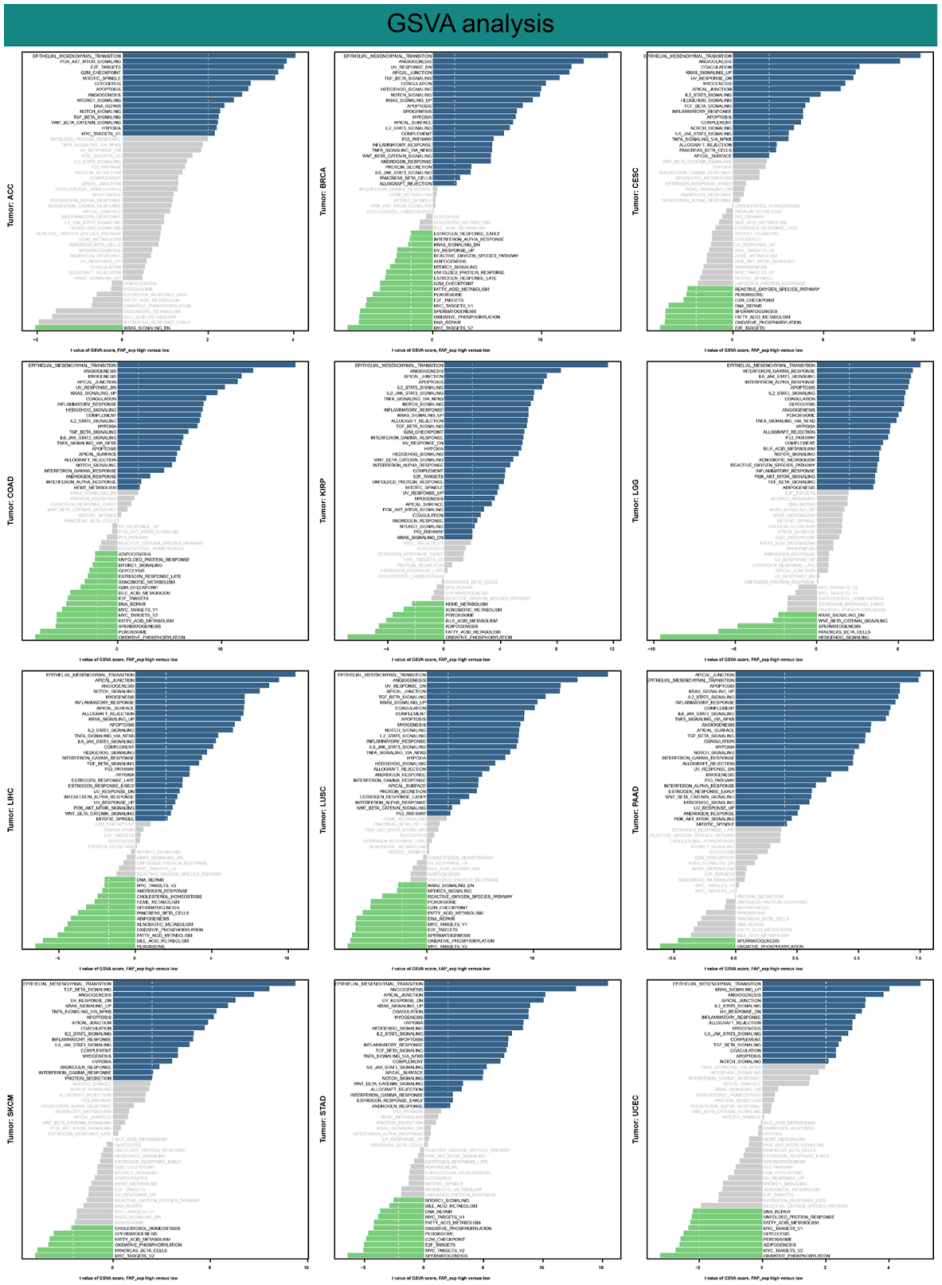
Results of GSVA.

GSVA analysis provided further insights into the differences in pathway activity scores between groups with high and low FAP expression. Figure 12 confirms that patients with high FAP expression exhibit enhanced activity in the EMT process, angiogenesis, inflammatory response, hypoxia, apoptosis, and activation of key oncogenic pathways, including TGFβ, KRAS, Hedgehog, Notch, and Wnt/β-catenin pathway. Conversely, low FAP expression is predicted to negatively regulate pathways associated with spermatogenesis, DNA repair, and energy metabolism, including oxidative phosphorylation, glycolysis, bile acid metabolism, and fatty acid metabolism.

### Immunotherapy prediction and drug sensitivity analysis

**Figure 13:**
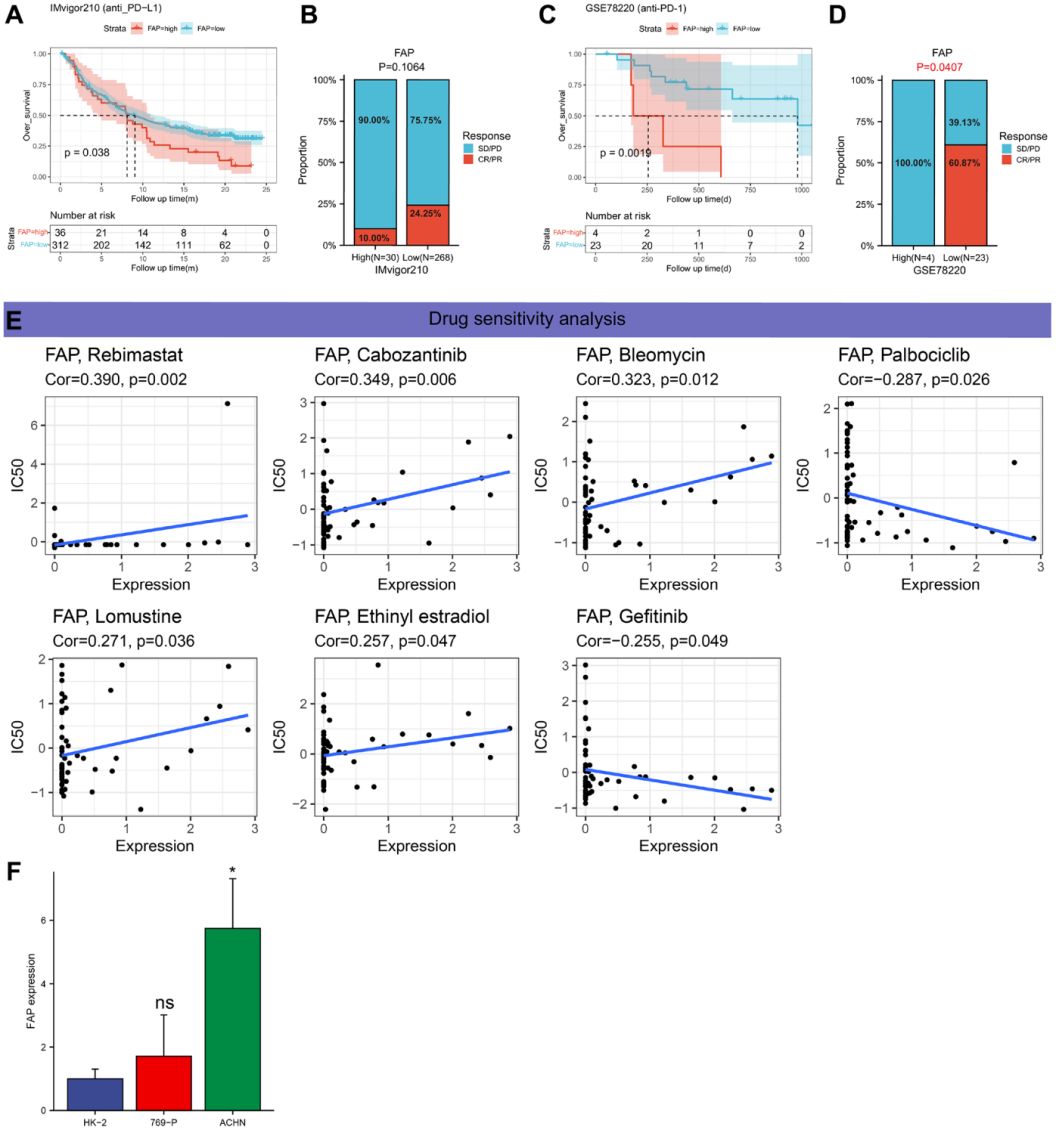
**Immunotherapy prediction analysis and drug sensitivity analysis.** (**A**) Kaplan-Meier analysis of the association between FAP expression and OS in the IMvigor210 cohort. (**B**) The proportion of BLCA patients who responded to anti-PD-L1 therapy in the groups with the low and high FAP expression. (**C**) Kaplan-Meier analysis of the association between FAP expression and OS in the GSE78220 cohort. (**D**) The proportion of SKCM patients who responded to anti-PD1 therapy in the groups with the low and high FAP expression. (**E**) An illustration of the relationship between FAP expression and expected medication response. (**F**) The mRNA expression levels of FAP in different cell lines (HK-2, 769-P, ACHN) were measured by RT-qPCR.

There is substantial evidence indicating that immunotherapy, specifically ICIs, can significantly improve the survival outcomes of patients with tumors [30–32]. This study assesses the predictive role of FAP expression in determining the response to immunotherapy among tumor patients treated with ICIs. The KM survival analysis demonstrates a correlation between increased FAP expression and poorer clinical outcomes in SKCM and BLCA (Figure 13A, 13C). In the IMvigor210 cohort (BLCA), patients with high FAP expression exhibited an anti-PD-L1 response rate of 10.00%, which was significantly lower than the 24.25% rate observed in patients with low FAP expression (Figure 13B). However, the lack of statistical significance in the Chi-square test may be attributed to the small sample size. Similarly, within the GSE78220 cohort (SKCM), patients with high FAP expression exhibited a 0% response rate to anti-PD-1 therapy, whereas 60.87% of patients with low FAP expression responded positively (Figure 13D). These findings suggest that FAP expression can serve as a potential immunotherapy biomarker for predicting the response rate among SKCM patients undergoing ICI treatment. Furthermore, FAP expression was found to be positively associated with drug response in patients treated with Rebimastat, Cabozantinib, Bleomycin, Lomustine, and Ethinyl estradiol, while anticancer drugs Gefitinib and Palbociclib showed a negative association with FAP expression (Figure 13E).

### RT-qPCR

To validate the expression levels of FAP mRNA, RT-qPCR was conducted in KIRC cells and normal cell lines (Figure 13F). The results demonstrated higher FAP expression in ACHN cells compared to HK-2 cells. However, no significant difference was observed between 769-p cells and HK-2 cells. Overall, the experimental results align with the bioinformatics analysis results obtained from the TCGA data.

## DISCUSSION

Compared to normal tissues, FAP is upregulated in 22 tumors and downregulated in 6 tumors. FAP expression was significantly lower in tumor tissues of CESC, SKCM, KICH, THCA, UCEC, and UCS compared to their respective matched-normal tissues. Cox regression models demonstrated no correlation between FAP expression and the prognosis of the aforementioned six tumors. However, the KM survival analysis revealed that high FAP expression is associated with a shorter DSS in UCEC. Notably, FAP expression was relatively low in KICH, whereas it was significantly higher in KIRP and KIRC compared to normal kidney tissue. High FAP expression was associated with shorter survival in KIRP and KIRC, potentially attributed to variations in primary tumor location.

RT-qPCR results indicated significantly higher expression of FAP in ACHN cells compared to HK-2 cells. Furthermore, our study confirmed that high FAP expression is associated with a poorer prognosis in most cancers. However, it is linked to a better prognosis in UVM, DLBC, and PCPG. It is worth noting that the role of FAP in UVM, DLBC, and PCGP has not been elucidated, warranting further investigation.

FAP, being a specific marker of tumor-associated fibroblasts, demonstrates variable expression levels across different cancer types and predicts diverse, and at times contradictory, prognoses among cancer patients. Various factors, including genetic and epigenetic alterations, TME, and signaling pathways implicated in cancer progression, can influence the expression of FAP, providing a potential explanation for this phenomenon. Each cancer type possesses unique molecular characteristics and a distinct TME, which can contribute to the variations in FAP expression. For example, different cancer types may originate from diverse cell lineages, harbor varying mutational landscapes, or display heterogeneous immune responses. These factors can affect the activation of fibroblasts and the expression of FAP in the TME. Furthermore, distinct signaling pathways and transcription factors that are active in each cancer type can regulate the expression of FAP.

Moreover, the effect of FAP expression on patient survival may vary across different cancer types. This discrepancy can be attributed to various factors, such as the interplay between FAP-expressing CAFs and tumor cells, the immunosuppressive effects of FAP, and the overall composition and dynamics of the TME. Investigating the differential expression of FAP in diverse cancer types and its implications for patient survival would yield valuable insights into the underlying biology and clinical significance of FAP in cancer.

We observed a significant correlation between FAP elevation and tumor volume, as well as the depth of tumor infiltration, in BRCA, COADREAD, GBMLGG, STES, STAD, THYM, ACC, and SKCM. Studies have reported that the FAP inhibitor talabostat significantly inhibits tumor growth in patients with early-stage COAD but shows limited efficacy in patients with advanced-stage COAD. Our findings showed that patients with T1-staged COADREAD have higher FAP expression compared to those in T3 and T4 stages. Therefore, patients with advanced COAD may exhibit lower FAP expression, impairing the treatment efficacy of talabostat.

Regarding the connection between FAP expression and pathological stage across different tumors, our findings demonstrated that in STAD and THYM, FAP expression is higher in stage I and II than in stage III and IV. In UCEC, FAP expression is higher in stage I than in stage II, consistent with previous studies. These results suggest that FAP can serve as a biomarker for patients with tumors at specific pathological stages. Furthermore, FAP expression is closely associated with DNA methylation. High FAP methylation levels correlate with better survival in STAD, HNSC, SARC, and LIHC, while they are associated with worse survival in TGCT, SKCM, LUSC, and PAAD. ROC curves demonstrate that FAP expression has higher predictive power in fifteen tumors, indicating its potential as a diagnostic biomarker. Notably, the tumors with an AUC > 0.9 were CHOL, DLBC, GBMLGG, HNSC, OSCC, PAAD, STAD, THYM, UCEC, and UCS, separately.

In terms of the correlation between FAP expression and the TME, ESTIMATE analysis reveals a significant positive connection between FAP expression and StromalScore in 33 types of cancer, ImmunScore in 28 types of cancer, and ESTIMATEScore in 32 types of cancer. These findings suggest that FAP participates in the malignant progression of tumors by influencing the TME.

TIICs play a critical role in tumor progression, which is closely associated with the prognosis of tumor patients and the immune response [33]. Previous studies have reported that upregulation of FAP induces immunosuppression by increasing the infiltration of immune-suppressive cells [34]. Our findings confirm that FAP expression exhibits a negative association with CD8 T cells, monocytes, and activated dendritic cells, while it shows a positive correlation with M0, M1, and M2 macrophages in the majority of tumors. Furthermore, the enrichment analysis demonstrates that FAP may influence tumorigenesis through the regulation of various cellular processes, including cell proliferation, migration, EMT, energy metabolism, immunoglobulin synthesis and transport, and B/T cell-mediated immunity. These results are consistent with previous studies.

The role of FAP expression in tumor immunotherapy was investigated using the IMvigor210 cohort (BLCA) and the GSE78220 cohort (SKCM). The results indicate that high FAP expression is associated with shorter survival and lower sensitivity to immunotherapy responses in SKCM. However, FAP expression showed no correlation with immunotherapy response in BLCA, potentially due to the limited sample size. Therefore, our results suggest that FAP could serve as a potential predictor for the response to immunotherapy. Additionally, FAP expression exhibits a positive correlation with the IC50 values of Rebimastat, Cabozantinib, Bleomycin, Lomustine, and Ethinyl estradiol, whereas it displays a negative correlation with the IC50 values of Gefitinib and Palbociclib.

In summary, the analysis of FAP expression across various cancer types revealed a strong association between FAP upregulation and clinical outcomes, tumor diagnosis, DNA methylation levels, and immunotherapy responses. FAP could serve as a potential biomarker for diagnosis, prognosis, and prediction of the response to immunotherapy. Additionally, FAP contributes to tumorigenesis and tumor immunity by modulating the infiltration of immune cells. This study elucidates the role of FAP in tumor development and provides a valuable reference for targeting FAP to enhance immunotherapy.

## Supplementary Materials

Supplementary Figures

Supplementary Table 1

Supplementary Table 2
