# Supplementary material for: Identifying the oncogenic roles of FAP in human cancers based on systematic analysis
Source: Aging (Albany NY). 2023 Jul 24;15(14):7056–83. doi: 10.18632/aging.204892 (PMC10415543; doi:10.18632/aging.204892)
Supplement: Supplementary Figures [file aging-15-204892-s001.pdf]

SUPPLEMENTARY FIGURES

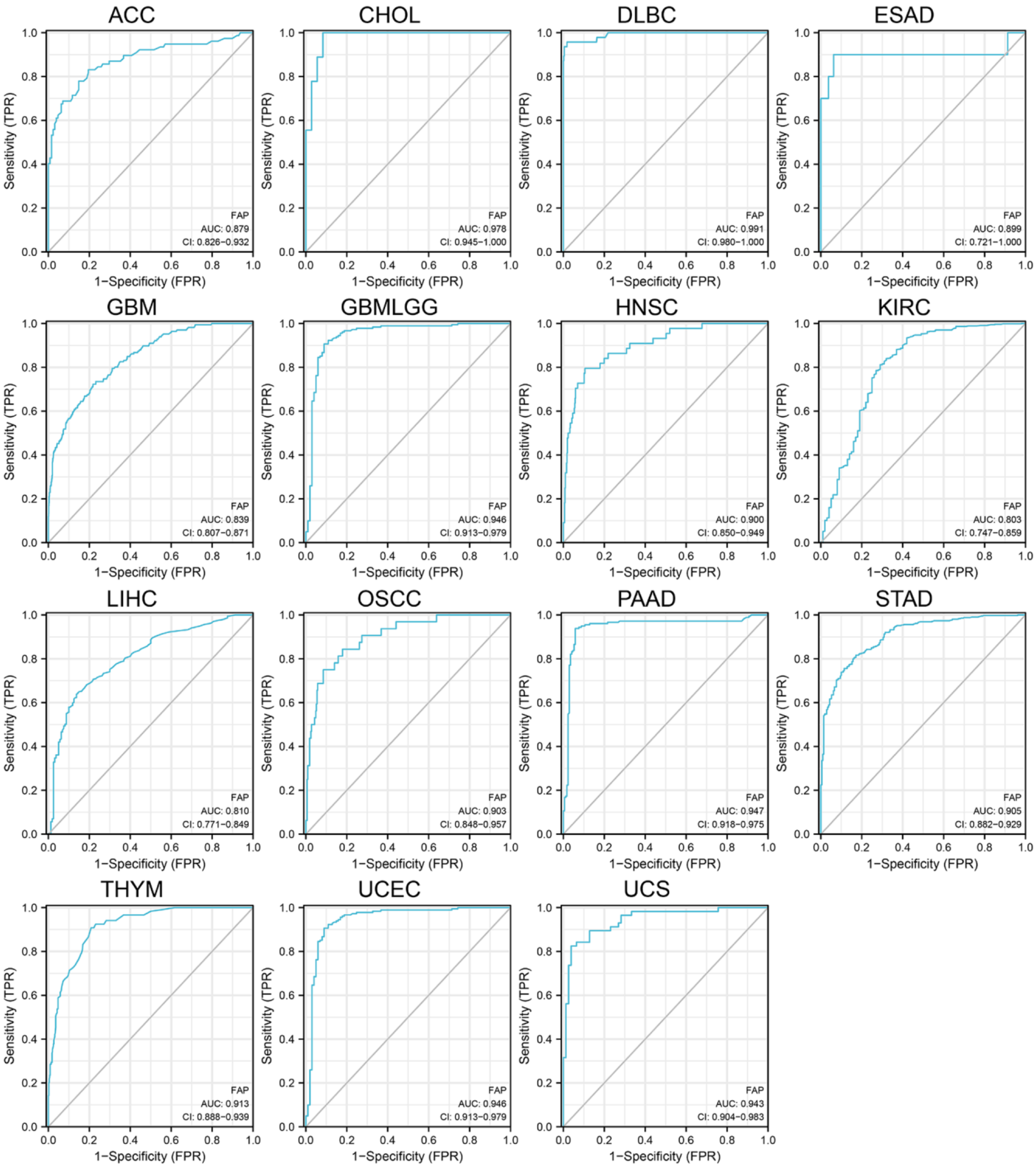

Supplementary Figure 1. ROC curves for FAP in adrenocortical carcinoma (ACC), cholangiocarcinoma (CHOL), lymphoid neoplasm diffuse large b-cell lymphoma (DLBC), esophageal adenocarcinoma (ESAD), glioblastoma multiforme (GBM), glioma (GBMLGG), head and neck squamous cell carcinoma (HNSC), kidney renal clear cell carcinoma (KIRC), liver hepatocellular carcinoma (LIHC), oral squamous cell carcinoma (OSCC), pancreatic adenocarcinoma (PAAD), stomach adenocarcinoma (STAD), thymoma (THYM), uterine corpus endometrial carcinoma (UCEC), uterine carcinosarcoma (UCS).

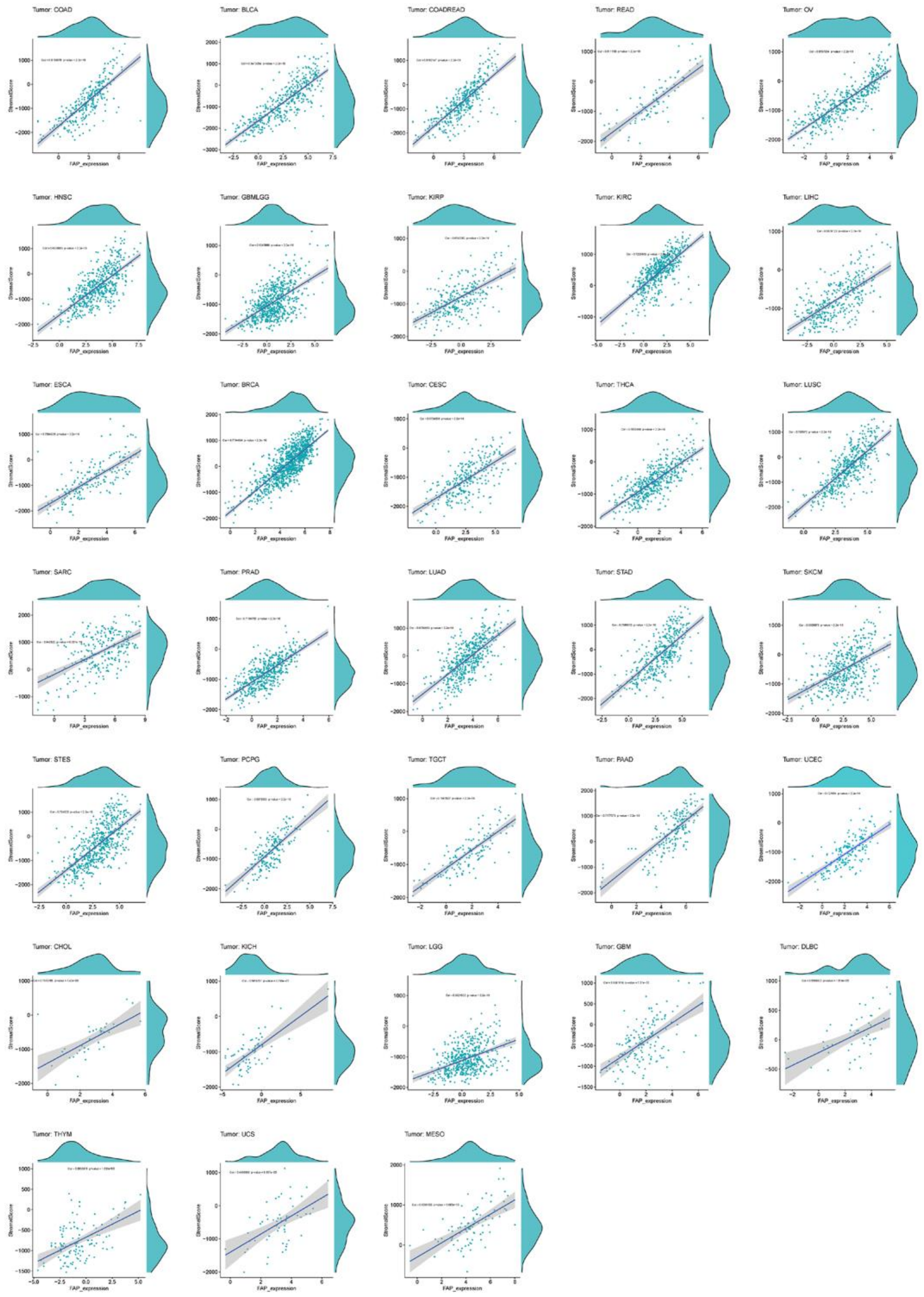

Supplementary Figure 2. Correlation between FAP and stromal scores in pan-cancer.

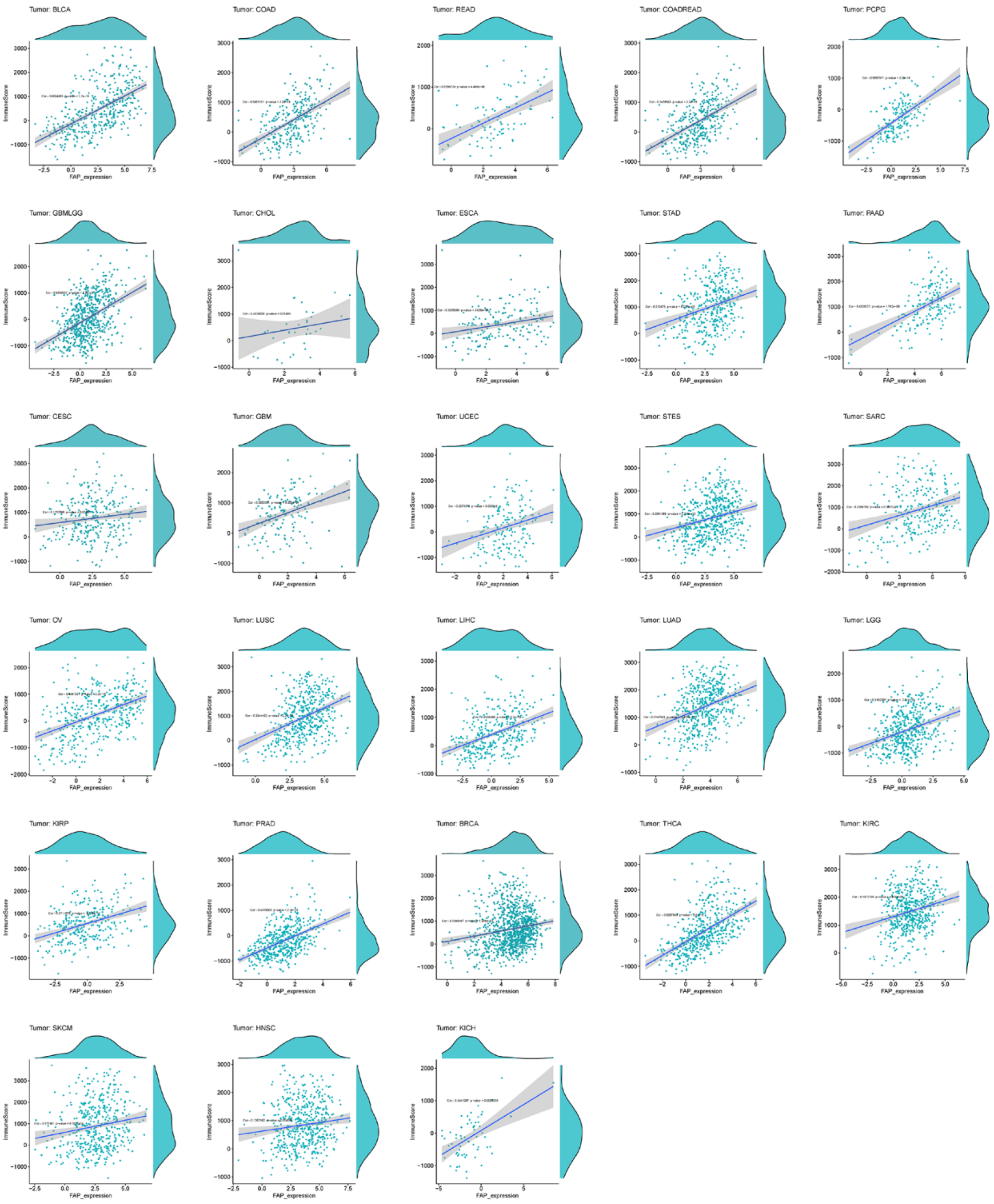

Supplementary Figure 3. Correlation between FAP and immune scores in pan-cancer.

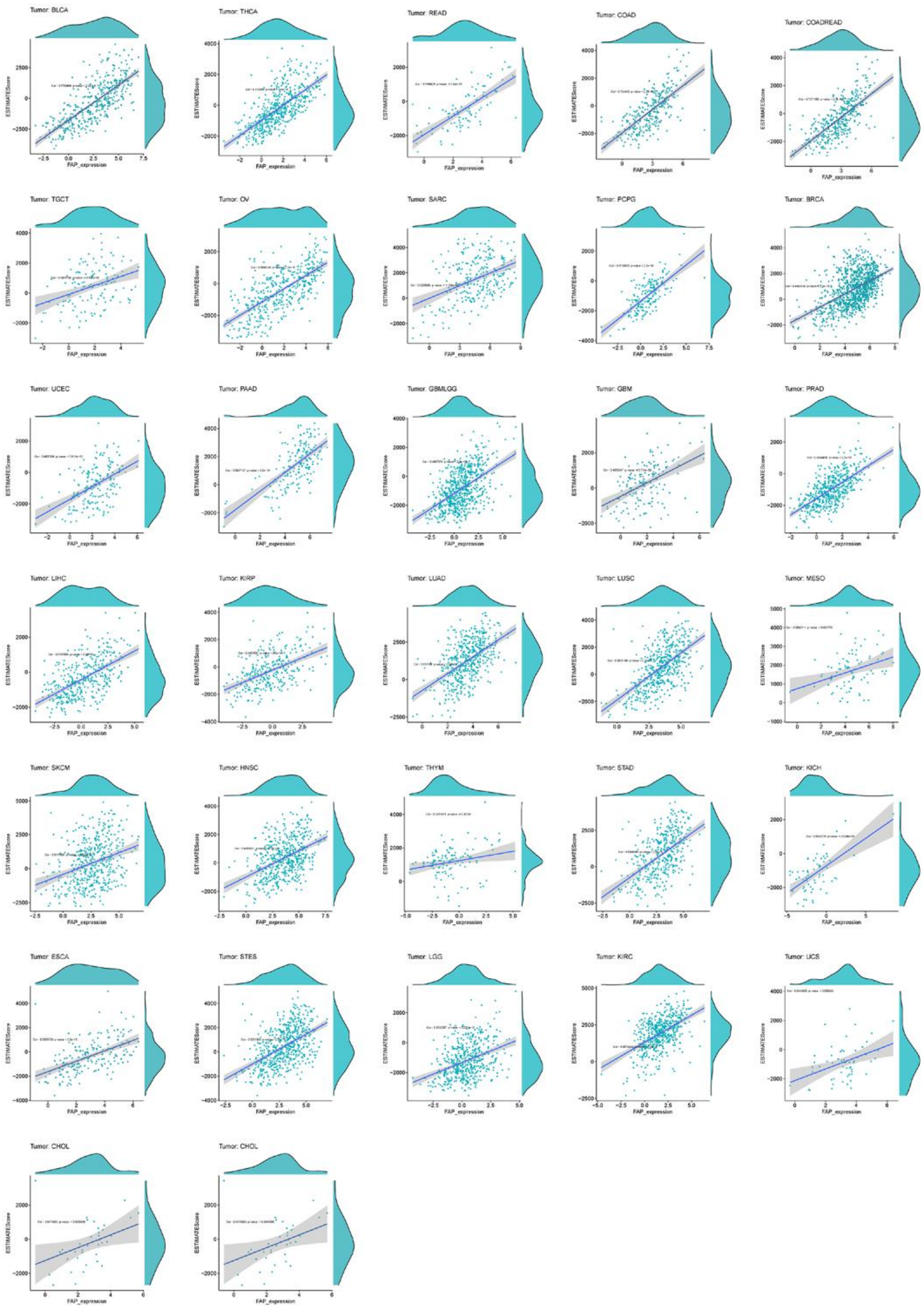

Supplementary Figure 4. Correlation between FAP and ESTIMATES cores in pan-cancer.

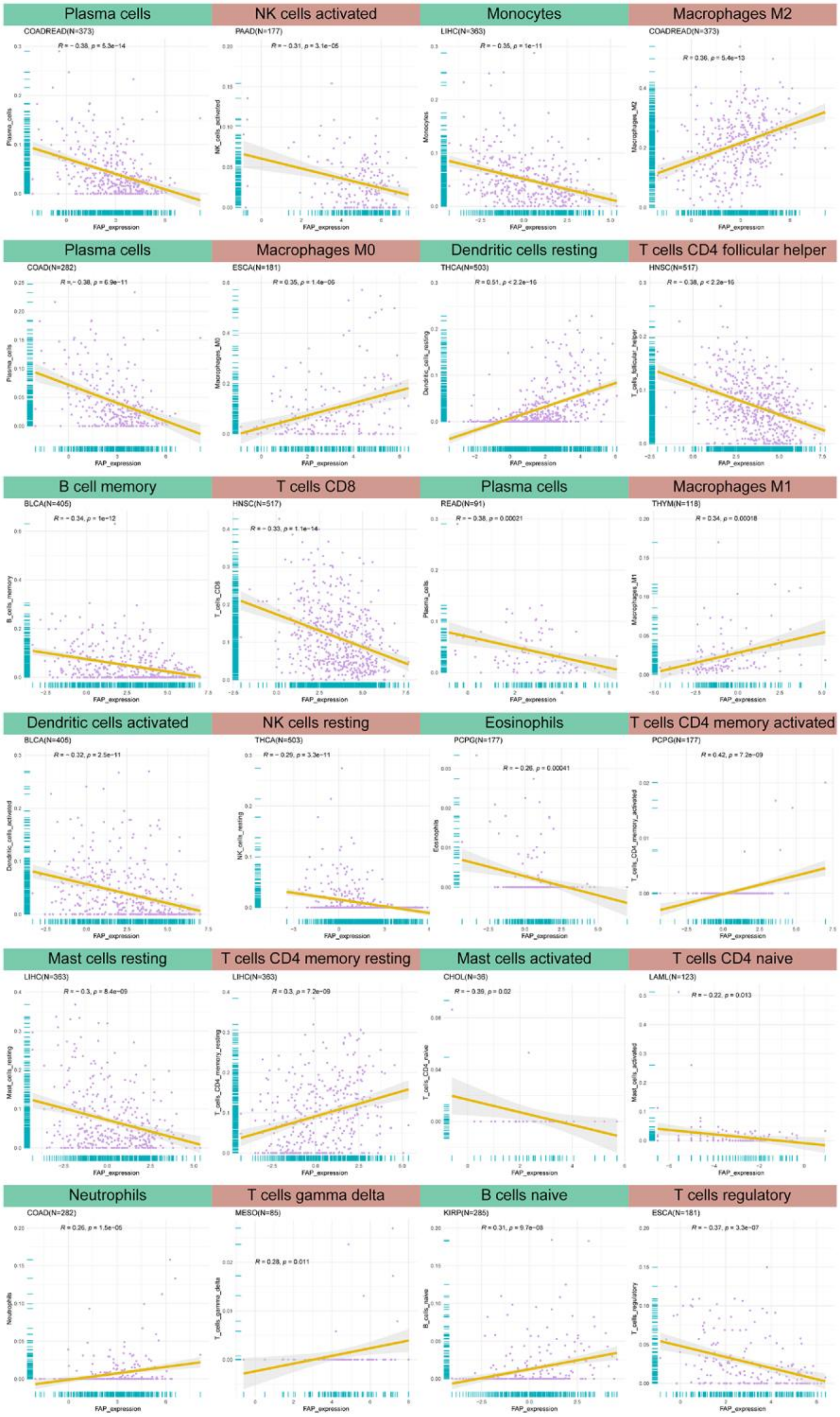

Supplementary Figure 5. Relationship between FAP expression and the infiltration scores of 22 immune cell types.

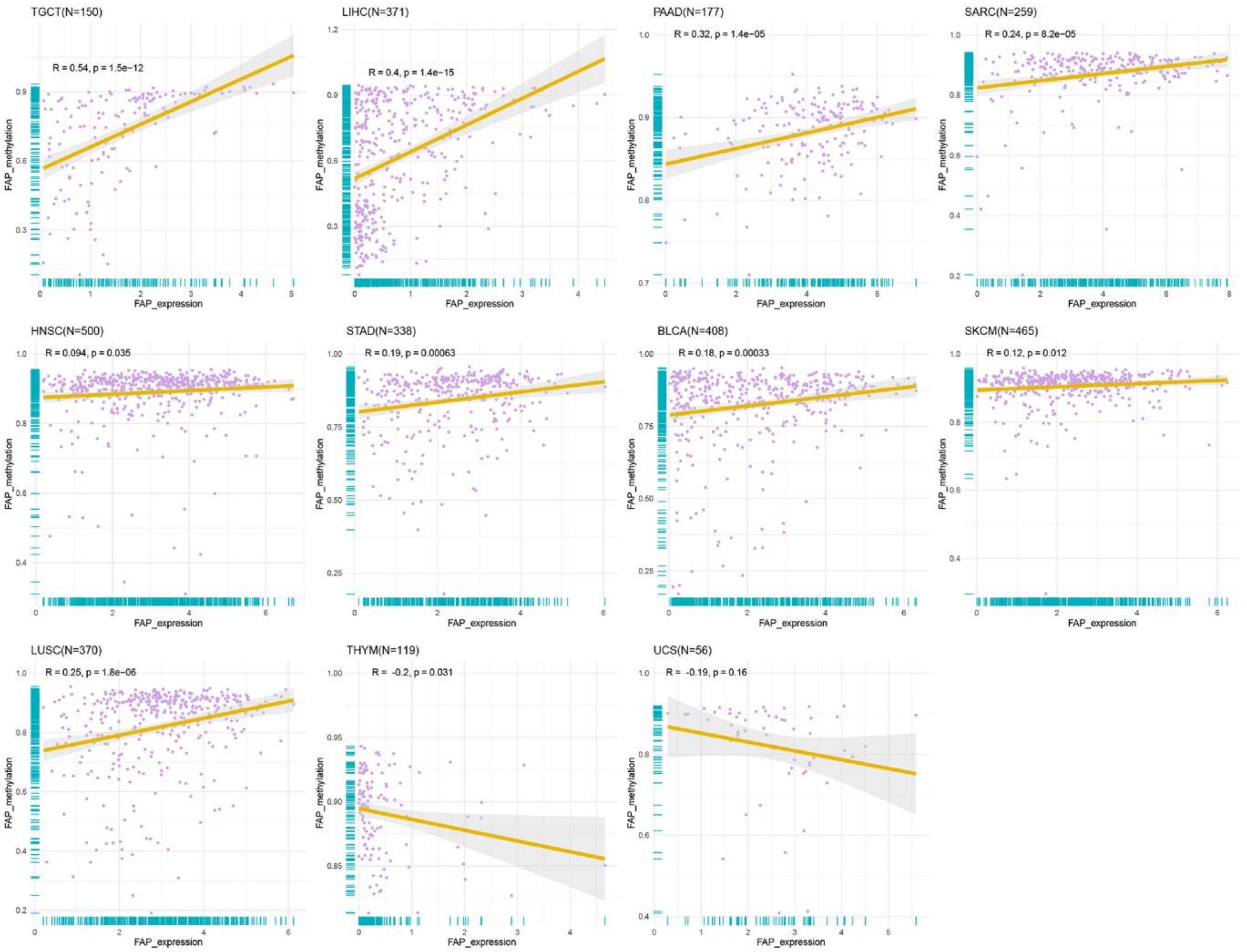

Supplementary Figure 6. Correlation between FAP expression and gene promoter methylation in testicular germ cell tumors (TGCT), liver hepatocellular carcinoma (LIHC), pancreatic adenocarcinoma (PAAD), sarcoma (SARC), head and neck squamous cell carcinoma (HNSC), stomach adenocarcinoma (STAD), bladder urothelial carcinoma (BLCA), skin cutaneous melanoma (SKCM), lung squamous cell carcinoma (LUSC), thymoma (THYM), uterine carcinosarcoma (UCS).
