# Supplementary material for: Identifying the oncogenic roles of FAP in human cancers based on systematic analysis
Source: Aging (Albany NY). 2023 Jul 24;15(14):7056–83. doi: 10.18632/aging.204892 (PMC10415543; doi:10.18632/aging.204892)
Supplement: Supplementary Table 1 [file aging-15-204892-s002.pdf]

SUPPLEMENTARY TABLE

Supplementary Table 1. Relationship between FAP expression and immune cell infiltration in eight cancers.

Cell type	BRCA (*N* = 1077) (*P*-value/Cor)	THCA (*N* = 503) (*P*-value/Cor)	BLCA (*N* = 405) (*P*-value/Cor)	PRAD (*N* = 495) (*P*-value/Cor)	THYM (*N* = 118) (*P*-value/Cor)	OV (*N* = 416) (*P*-value/Cor)	LUSC (*N* = 491) (*P*-value/Cor)	COADREAD (*N* = 373) (*P*-value/Cor)
Naive B cells	***/0.12	***/0.17	***/0.18	***/0.16	*/0.22	*/0.1	−0.03	0
Memory B cells	***/−0.16	*/0.09	***/−0.34	0.01	*/0.23	***/−0.21	**/−0.15	*/−0.1
Plasma cells	−0.03	*/0.09	−0.08	***/−0.16	***/0.32	0.04	**/−0.12	***/−0.38
CD8 T cells	**/−0.09	***/−0.27	−0.07	***/−0.2	−0.13	0.07	***/−0.23	***/−0.19
Naive CD4 T cells	***/−0.13	−0.07	***/−0.29	−0.07	***/−0.37	**/−0.14	0	−0.07
Resting CD4 memory T cells	***/0.21	**/0.12	**/0.14	***/0.26	0.17	***/0.25	***/0.4	***/−0.19
Activated CD4 memory T cells	***/−0.13	0.08	***/0.21	−0.01	***/0.35	*/0.12	0	*/−0.13
Follicular T helper cells	***/−0.29	−0.07	***/−0.34	***/−0.21	***/−0.35	***/−0.19	***/−0.37	***/−0.25
Regulatory T cells (Tregs)	−0.06	***/0.3	***/−0.26	***/0.18	−0.14	−0.04	0.05	−0.03
Gamma delta T cells	0.04	0.02	−0.09	−0.08	NA	0.01	−0.06	−0.06
Resting NK cells	***/−0.18	***/−0.29	−0.04	**/−0.14	0.14	−0.08	0.09	−0.04
Activated NK cells	***/−0.16	*/−0.09	0	−0.02	**/0.24	−0.05	***/−0.2	−0.1
Monocytes	**/−0.09	*/−0.1	***/−0.22	***/−0.16	−0.15	***/−0.27	***/−0.2	−0.06
M0 Macrophages	−0.02	0.06	***/0.28	−0.02	***/0.41	−0.02	***/0.24	***/0.23
M1 Macrophages	−0.05	***/0.19	***/0.33	*0.1	***/0.34	***/0.25	−0.01	**/0.17
M2 Macrophages	*/0.06	**/−0.12	***/0.34	***/0.21	***/0.32	*/0.11	0.07	***/0.36
Resting dendritic cells	*/0.07	***/0.51	−0.08	***/0.22	**/−0.24	**/0.14	−0.04	0
Activated dendritic cells	***/−0.14	*/0.09	***/−0.32	0.01	*/0.19	***/−0.2	*/−0.09	***/−0.17
Resting mast cells	***/0.15	***/−0.2	−0.01	***/−0.18	−0.01	***/−0.24	**/−0.13	*/0.12
Activated mast cells	−0.03	**/−0.14	0.01	−0.03	*/0.2	*/0.1	*/0.09	−0.05
Eosinophils	*/−0.06	***/−0.2	*/−0.11	*/−0.09	0.04	**/−0.16	**/−0.12	0.05
Neutrophils	***/0.13	−0.03	0.09	0.01	*/0.23	*/0.12	0.03	***/0.19
